# Nanostring-Based Multigene Assay to Predict Recurrence for Gastric Cancer Patients after Surgery

**DOI:** 10.1371/journal.pone.0090133

**Published:** 2014-03-05

**Authors:** Jeeyun Lee, Insuk Sohn, In-Gu Do, Kyoung-Mee Kim, Se Hoon Park, Joon Oh Park, Young Suk Park, Ho Yeong Lim, Tae Sung Sohn, Jae Moon Bae, Min Gew Choi, Do Hoon Lim, Byung Hoon Min, Joon Haeng Lee, Poong Lyul Rhee, Jae J. Kim, Dong Il Choi, Iain Beehuat Tan, Kakoli Das, Patrick Tan, Sin Ho Jung, Won Ki Kang, Sung Kim

**Affiliations:** 1 Division of Hematology-Oncology, Department of Medicine, Samsung Medical Center Sungkyunkwan University School of Medicine, Seoul, Korea; 2 Samsung Cancer Research Institute, Samsung Medical Center, Seoul, Korea; 3 Department of Pathology, Samsung Medical Center Sungkyunkwan University School of Medicine, Seoul, Korea; 4 Department of Surgery, Samsung Medical Center, Sungkyunkwan University School of Medicine, Seoul, Korea; 5 Department of Radiation Oncology, Samsung Medical Center, Sungkyunkwan University School of Medicine, Seoul, Korea; 6 Department of Gastroenterology, Samsung Medical Center, Sungkyunkwan University School of Medicine, Seoul, Korea; 7 Department of Radiology, Samsung Medical Center, Sungkyunkwan University School of Medicine, Seoul, Korea; 8 Cancer and Stem Cell Biology, Duke-NUS Graduate Medical School Singapore, Singapore; 9 Genome Institute of Singapore, Singapore, Singapore; 10 Cancer Science Institute of Singapore, Singapore, Singapore; University of Texas MD Anderson Cancer Center, United States of America

## Abstract

Despite the benefits from adjuvant chemotherapy or chemoradiotherapy, approximately one-third of stage II gastric cancer (GC) patients developed recurrences. The aim of this study was to develop and validate a prognostic algorithm for gastric cancer (GCPS) that can robustly identify high-risk group for recurrence among stage II patients. A multi-step gene expression profiling study was conducted. First, a microarray gene expression profiling of archived paraffin-embedded tumor blocks was used to identify candidate prognostic genes (N = 432). Second, a focused gene expression assay including prognostic genes was used to develop a robust clinical assay (GCPS) in stage II patients from the same cohort (N = 186). Third, a predefined cut off for the GCPS was validated using an independent stage II cohort (N = 216). The GCPS was validated in another set with stage II GC who underwent surgery without adjuvant treatment (N = 300). GCPS was developed by summing the product of Cox regression coefficients and normalized expression levels of 8 genes (LAMP5, CDC25B, CDK1, CLIP4, LTB4R2, MATN3, NOX4, TFDP1). A prospectively defined cut-point for GCPS classified 22.7% of validation cohort treated with chemoradiotherapy (N = 216) as high-risk group with 5-year recurrence rate of 58.6% compared to 85.4% in the low risk group (hazard ratio for recurrence = 3.16, p = 0.00004). GCPS also identified high-risk group among stage II patients treated with surgery only (hazard ratio = 1.77, p = 0.0053).

## Introduction

Gastric cancers are highly lethal malignancies with five-year survival rates being one of the worst reported for any solid tumors. According to data from the National Cancer Institute Surveillance, Epidemiology and End Results (SEER) Program, the five-year survival for patients with gastric cancer (GC) improved only modestly over the last 50 years, from 12 to 22 percent [1]. The propensity of GC for early metastatic dissemination has been well documented in previous studies [2], [3]. Based on the recent adjuvant phase III trials, survival benefit from adjuvant chemotherapy or chemoradiation therapy has been documented in GC [4]–[7]. However, 25 to 40% of all surgically resected GC patients still develop recurrences that are not amenable to re-resection [4], [7]–[9]. For pathologic stage III and IV GC, 5-year disease-free survival rates are very poor (stage IIIA, 57.6%, stage IIIB, 39.6%; and stage IV 26.3%) [8] implicating that these tumors have inherently aggressive behaviour. In contrast, pathologic stage II GC patients have more favorable clinical outcome with 5-year disease free survival rates of 76% −90% following surgery and adjuvant treatment [8], [9]. Nevertheless, there is a wide spectrum of clinical aggressiveness even within the same stage with some patients being cured with surgery alone while some patients recur shortly after surgery and adjuvant chemoradiation therapy. Hence, based on the hypothesis that there is a significant molecular heterogeneity, we designed a large-scaled gene expression profiling study to develop a molecular test which may efficiently discriminate low-risk from high-risk GC groups for recurrence after surgery.

A molecular test that identifies high-risk patients for recurrence may lead to optimized perioperative treatment strategies in GC. The discovery phase included GC patients from all clinical stages treated with chemoradiotherapy (N = 520). Tumor blocks from these patients were subjected to prognostic gene discovery using Whole Genome DASL assay (WG-DASL) (Illumina, San Diego, CA), a microarray gene expression profiling method for formalin-fixed paraffin-embedded tissue (FFPE). The purpose was to develop hypothesis for clinical utility and discover candidate prognostic or internal reference genes that will help design focused gene expression assay. Results from this phase suggested that clinical utility of a gene expression based prognostic algorithm may potentially distinguish a high-risk group among stage II patients.

The objective of the project was to develop a hypothesis with clinical utility and discover candidate prognostic or internal reference genes in order to design focused gene expression assays. The results from the discovery phase suggest that clinical utility of a gene expression-based prognostic algorithm may potentially distinguish a high-risk group among stage II patients. With the use of a robust multistep prognostic algorithm, Gastric Cancer Prognostic Score (GCPS) for stage II GC patients was developed to identify high-risk patients for recurrence after surgery.

## Methods

**Figure 1:**
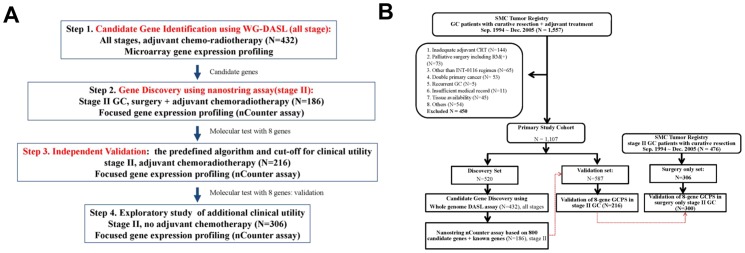
The Consort Diagram. (A) A multi-step approach gene expression profiling design, (B) Consort Diagram.

From September 1994 to December 2005, 1,557 GC patients underwent curative gastrectomy at Samsung Medical Center. Among those, 1,107 patients were selected based on following criteria: histologically confirmed adenocarcinoma of the stomach; surgical resection of tumour without macroscopic or microscopic residual disease; age ≥18; pathology stage IB (T2bN0, T1N1 but not T2aN0) to IV, according to the American Joint Committee on Cancer (AJCC) staging system (6^th^ Ed); complete surgical record and treatment record, and patients receiving the INT-0116 regimen as adjuvant treatment [7]. The study was approved by the institutional review board of the Samsung Medical Center, Seoul, South Korea (IRB approval number: SMC 2010-10-025). All study participants provided written informed consent form recommended by the IRB. In the patients who have deceased at the time of study entry, written informed consent forms were waived by the IRB. Study design and patient cohorts are provided according to REMARK guideline (**Figure 1A, 1B**, **File S1, Section 1**). Of the cohort of 1,107 patients, a discovery set of 520 patients and a validation set of 587 patients were randomly assigned and allocated to 6 batches stratified by tumor size and year of surgery for WG-DASL assay.

To avoid false-positive conclusions due to over-fitting, prognostic algorithms and their predefined cut-points were tested in independent cohorts that were not used for prognostic gene discovery and algorithm building. A 4-phase study was designed, with 4 pre-defined independent cohorts recruited from the Samsung Medical Center. The first 3 cohorts include patients with similar clinical and pathological features from chemoradiotherapy-treated study cohorts (**File S1, Section 2**). The **first phase (discovery phase)** of the study included GC patients from all clinical stages who were treated with chemo-radiotherapy (N = 520) [8]. Tumor blocks from these patients were subjected to prognostic gene discovery using the WG-DASL (Illumina, San Diego, CA), a microarray gene expression profiling method for FFPE [7]. An ad-hoc external validation of the gene set was performed to minimize any bias from single institutional cohort. The **second phase (algorithm development)** was to translate findings from the first phase into a clinically applicable test format. We chose the nCounter platform (Nanostring Technologies, Seattle, WA), because of its ability to interrogate the expression levels of up to 800 genes using total RNA extracted from FFPE in a single-tube reaction [8]. We screened stage II patients from the first phase (N = 186) for *de novo* discovery of prognostic genes, selected ideal combinations of genes using the gradient least absolute shrinkage and selection operator (LASSO) algorithm [10], and then built a first-generation GCPS (GCPS-g1) by adding the products of normalized gene expression and coefficients from the Cox model for DFS. In the **third** cohort of stage II patients (N = 216). In the **fourth phase (testing of clinical utility in a surgery-only setting)**, we tested the potential clinical utility of GCPS in stage II patients treated with surgery only. A time stamp protocol (**Figure S12**) was developed before processing of this final cohort. We subsequently developed a refined second-generation GCPS (GCPS-g2) (the final gene set) by analyzing the combined stage II cohorts from the second and third phases of the study.

### Gene expression profiling using whole genome-DASL assay

Before each gene profiling experiment, tissue samples were randomly allocated to different batches stratified by surgery time (before 2000 vs. after 2000) and tumor size (≤5 cm vs >5 cm) to minimize any variations from the DNA quality. Total RNA was extracted from 2–4 sections of 4-µm thick FFPE sections from representative primary tumor blocks using the High Pure RNA Paraffin kit (Roche Diagnostic, Mannheim, Germany) after removing non-tumor elements by manual macrodissection guided by hematoxylin and eosin stained slides. WG-DASL assay was performed using 200 ng of RNA following the manufacturer's instruction [11]. For nCounter assay, 200 ng of total RNA was hybridized with the custom designed code set of 800 genes for 18 hours at 65°C and processed according to manufacturer's instruction [12]. The data were normalized to average expression levels of 48 internal reference genes selected from microarray experiment. The detailed description of the discovery phase using WG-DASL assay is provided in **File S1, Section 3**. The comparability in DNA quality of FFPE tissue and fresh frozen tissues using DASL assay was published previously [13], [14].

### Prognostic model building and validation

The algorithm for n-Counter-based assay development for clinical utility based on WG-DASL is provided in **File S1, Sections 4–6**. We used the gradient lasso algorithm to fit a prediction model based on Cox's proportional hazards model for DFS using the probes with marginal p value<0.01 (**Figure S2 in File S1**) [10]. We used leave one out cross validation with de novo discovery at each leave one out step to assess the performance of the prognostic model within the discovery cohort. Optimal cut-point was determined by creating a plot for p-values for each cut-point for the prognostic score. For validation study, a priori defined algorithm and cut-point values were used. The validation method for GCPS is outlined in **File S1, Section 7**.

## Results

### Microarray gene expression profiling of GC patients treated with adjuvant chemoradiotherapy (phase 1)

**Figure 2:**
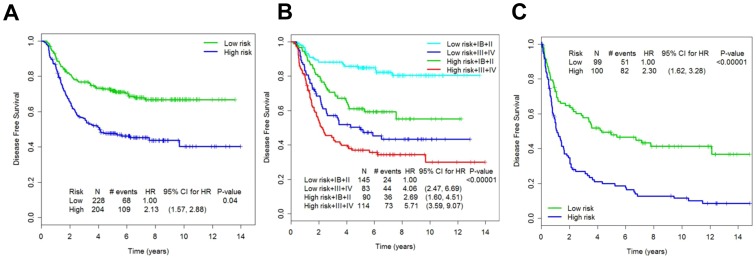
Kaplan-Meier curves for high risk and low risk groups classified by leave one out cross validation procedure. HR denotes hazard ratio and p-value is calculated from 100 permutations. (A) all stage patients, high vs low risk; (B) according to stage and risk groups; (C) External validation of the initial gene expression profiling.

We performed gene expression profiling of FFPE from the discovery cohort of 520 cases of stage IB–IV GC treated with standard chemoradiotherapy after curative resection using the WG-DASL assay (**Figure 1**). Among them, 432 samples passed RNA quality control (GEO database GSE 26253) (**File S1, Section 3**). The primary end point was DFS. Univariate analysis identified 369 probes that were significantly associated with disease-free survival at p<0.01 without adjustment for other clinical variables (**File S1, Section 3d**). Next, gradient Lasso was used to develop a prognostic algorithm to predict recurrence (**File S1, Section 3e**). The leave-one-out cross-validation (LOOCV) procedure with *de novo* discovery of prognostic genes and the building of a prognostic algorithm at each step was used to examine the robustness of the prognostic algorithm. According to prognostic gene signatures (26 genes, **File S1, Section 3f**) and pathologic stages (localized vs. advanced), 432 patients were categorized into the following groups: low-risk and stage IB/II (N = 145; 5-year DFS, 84.8%), high-risk and stage IB/II (N = 90; 5-year DFS, 61.1%), low-risk and stage III/IV (N = 83; 5-year DFS, 48.9%), and high-risk and stage III/IV (N = 114; 5-year DFS, 36.9%) (**Figure 2**). As an ad-hoc analysis, we tested this gene signature using gene expression profiling data from the Singapore patient cohort (N = 199) to minimize any inherent bias from a single institution cohort [15]. In the external patient cohort, the gene signature was able to separate the high-risk group (N = 100) from the low-risk group (N = 99) for recurrence with statistical significance (p<0.00001; hazard ratio (HR), 2.3; 95% CI, 1.62–3.28) (**Figure 2**). These data suggest the main clinical utility of gene expression profiling of GC in the identification of high-risk patients among stage II patients (low vs. high risk stage IB/II, 84.8% vs. 61.1%; low vs. high risk stage III/IV, 48.9% vs. 36.9%). Therefore, for the development of clinical assay and validation, we focused on developing a gene-set which can robustly predict recurrence in stage II patients.

### Development of Gastric Cancer Prognostic Score (GCPS) for stage II GC using the nCounter assay (phase 2)

We custom designed an nCounter probe set comprised of candidate prognostic genes from WG-DASL microarray data (phase 1), as well as known cancer genes, kinase genes, and G protein-coupled receptor genes. To address the variability problem in the integrity of RNA molecules in archived FFPE due to pre-analytical variables, such as fixation time and age of the blocks, we applied within-sample normalization using a set of 48 internal reference genes selected from microarray data based on minimum variation across cases and lack of association with prognosis (**File S1, Section 4**). Correlation between hazard ratios of prognostic genes based on nanostring and WG-DASL are provided in **File S1, Section 4b**, and **Figure S3 in File S1**.

We profiled 186 stage II patients from the discovery set. After assessing the robustness of prognostic algorithms built by gradient LASSO through LOOCV, we applied gradient LASSO to all 186 patients and identified 8 genes (LAMP5, CDC25B, CDK1, CLIP4, LTB4R2, MATN3, NOX4, and TFDP1) that in combination provided robust prognostic information (**Table 1**). The GCPS was then developed as a linear combination of the Cox regression estimates and normalized expression levels of these 8 genes. The cut-point analysis demonstrates that the GCPS was most robust in identifying 25% of patients with worst outcomes (**File S1, Section 4**). We chose a cut-point of 0.2205 for prospective validation in the independent validation cohort.

**Table 1:** List of genes that constitute Gastric Cancer Prognostic Score and their Cox regression estimates used to calculate the score.

Gene Symbol	Chromosomal location	Gene Name	Cox regression estimate
LAMP5(BAD-LAMP, C20orf103)	20p12	Lysosomal-associated membrane protein family, member 5	0.0636
CDC25B	20p13	Cell division cycle 25 homolog B (S. pombe)	−0.0175
CDK1	10q21.1	Cyclin-dependent kinase 1	−0.1005
CLIP4 (UBASH3A, TULA, STS-2, TULA-1)	2p23.2	CAP-GLY domain containing linker family, member 4; Suppressor of T cell receptor signaling-2	0.4822
LTB4R2	14q11.2-q12	Leukotriene B4 receptor	−0.3950
MATN3	2p24-p23	Matrillin 3	0.2982
NOX4	11q14.2-q21	NADPH oxidase 4	0.0288
TFDP1	13q34	Transcription factor Dp-1	−0.2886

### Validation of GCPS and its predefined cut-point in stage II GC patients treated with chemoradiotherapy (phase 3)

**Figure 3:**
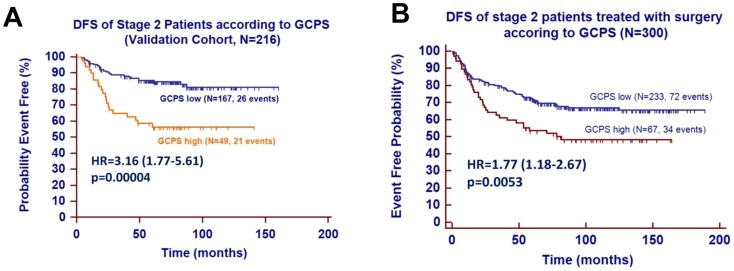
(A) DFS of stage 2 patients according to GCPS. (B) DFS of stage 2 patients treated with surgery according to GCPS.

To avoid the potential over-fitting issue associated with cross-validation [16], we validated GCPS with the fixed algorithm and cut-points in an independent patient cohort that was not used in gene discovery. The clinical and pathological features of 216 stage II patients from the validation set were similar to those from the discovery cohort (**File S1, Section 6**, and **Figures S4–6 in File S1**). When we applied GCPS-g1 to the validation set, the risk score distribution was very similar, suggesting the robust analytical performance of the assay (**File S1, Section 7a**, and **Figure S7 is File S1**). The predefined cut-point (0.2205) for GCPS-g1 classified 22.7% of tumors from the validation set as the high-risk group. The Kaplan Meier estimate of 5-year DFS for the high-risk patients was 58.6%, compared to 85.4% for the low-risk patients (HR for recurrence, 3.16; p = 0.00004) (**Figure 3**). GCPS was significant in both intestinal- and diffuse- type GCs, as shown in Figure S8 in File S1 (**File S1, Section 7b**). The multivariate analysis further shows that GCPS-g1 provided additional prognostic information, besides other known factors such as Lauren classification, differentiation grade, age, and surgery type (HR, 3.027; p = 0.00016; **Table 2**). Therefore, GCPS may be used to identify stage II patients who remain at high risk even after standard adjuvant chemoradiotherapy and who have similar risk of recurrence as stage III patients.

**Table 2:** Multivariate Cox regression analysis results on the validation set (N = 216).

	Hazard ratio	P-value
GCPS (low vs high-risk)	3.027	0.00016
LAUREN (intestinal vs diffuse)	0.541	0.18000
WHO (W/D∼M/D vs P/D*)	2.491	0.07600
AGE (65< = vs 65>)	1.496	0.19000
Surgery types (subtotal vs total gastrectomy)	1.121	0.71000

*W/D, well differentiated; M/D, moderately differentiated; P/D, poorly differentiated.

### GCPS as a prognostic factor for stage II GC patients treated with surgery only based on a prospectively designed protocol (phase 4)

The review of the clinical database identified 306 patients who did not receive postoperative treatment based on shared decision between physicians and patients (**File S1, Section 1, Table S1 in File S1**). These patients were subjected to exploratory analyses for assessing the prognostic role of GCPS in patients treated with surgery only and testing the hypothesis that the benefit from chemoradiotherapy is limited in high-risk patients defined by GCPS. For this step, we developed GCPS-g2 (**Table S8 in File S1**), the second-generation GCPS, by analyzing all stage II cases from phases 2 and 3 to maximize the sample size. We prospectively tested the predefined GCPS-g2 algorithm and cut-point as described in the time stamp protocol (**Figure S12 in File S1**). The GCPS-g2 predicted recurrence in 300 stage II tumors with a hazard ratio of 2.131 (95% CI, 1.428–3.180; p = 0.00021) (HR, 3.16) (**Figure S9 in File S1**). To minimize the possibility of over-fitting of the algorithm to chemoradiotherapy-treated patients, we also tested the GCPS-g1, which was previously validated in the chemoradiotherapy-treated cohort, in surgery alone cohort (**Figure S10 in File S1**). The hazard ratio for GCPS-g1 (HR, 1.77; 95% CI, 1.18–2.67; p = 0.0053) is similar to that for GCPS-g2 (**Figure 3**). Therefore, the GCPS robustly predicted recurrence in stage II GC with or without postoperative treatment. Based on this data, it can be speculated that high-risk stage II patients defined by GCPS did not gain a tremendous benefit from chemoradiotherapy.

### Expression of adverse prognostic genes in tumor microenvironments

In all 3 stage II cohorts included in this study, increased expression levels of 4 genes from GCPS (NOX4, LAMP5, MATN3, and CLIP4) were associated with poor prognosis. Since the known functions of these genes suggest their expression in microenvironments rather than actual tumor cells, we performed the nCounter assay for microdissected tumors versus stromal components from 4 representative high-risk tumors (**Figure S11 in File S1**). The expression of these genes was significantly higher in stromal components, compared to epithelial cancer cells, with NOX4 showing the most pronounced differences (p = 0.04).

## Discussion

In an initial discovery phase, we performed WG-DASL in all stage GC patients. Then, based on the WG-DASL data, we observed that the segregation of high risk group from low risk group was most significant in early stage Ib/II patients (low vs. high risk stage IB/II, 84.8% vs. 61.1%; low vs. high risk stage III/IV, 48.9% vs. 36.9%). Therefore, for the development of clinical assay and validation, we focused on developing a gene-set which can robustly predict recurrence in stage II patients. We developed and validated a prognostic algorithm for gastric cancer, GCPS, which can robustly identify high-risk groups for recurrence among stage II patients. GCPS, developed using the nCounter platform, showed the robust performance in FFPE samples. In addition, the inclusion of internal reference genes allowed the application of GCPS to individual patients. Therefore, we suggest that GCPS can be readily applied to routine clinical use. The GCPS of 8 genes (LAMP5, CDC25B, CDK1, CLIP4, LTB4R2, MATN3, NOX4, and TFDP1) were discovered and validated in over 700 stage II GC patients. We found that the GCPS identified high-risk GC patients for recurrence regardless adjuvant treatment and that high-risk stage II GC patients showed similar DFS to stage III patients. Notably, the GCPS predicted recurrence of both Lauren types (diffuse or intestinal) (**Figure S8 in File S1**).

Our data clearly demonstrate the presence of molecular heterogeneity in GC, which was associated with clinical outcomes but independent of clinicopathologic staging information. Our data indicate that stage IB/II patients had very poor prognosis when their tumors expressed poor-risk gene signatures. There was a difference of 23.7% in 5-year DFS between high-risk and low-risk gene signatures in stage IB/II patients, and 5-year DFS of high-risk stage IB/II patients was below 60%, despite the use of adjuvant chemoradiotherapy (**Figure 1**). Therefore, it may be necessary to prospectively design a trial to question whether chemoradiotherapy is required for stage IB/II patients with low-risk gene expression profiles. In order to minimize any potential bias from variations in clinical practice or surgery at a single center, we performed an ad hoc external validation of the signature to validate the signature. As shown in the Results, the signature consistently predicted recurrence in Singapore cohort.

Among the 8 final GCPS genes (LAMP5, CDC25B, CDK1, CLIP4, LTB4R2, MATN3, NOX4, and TFDP1), CDC25B and CDK1, which are known to be associated with cell proliferation, were found to correlate with favorable prognosis (negative Cox regression estimates in Table 2). Notably, a similar trend has been observed for colon cancer by a gene expression assay [17], [18]. These findings may reflect the differentiation status of these tumor cells, since normal gastric and colon mucosal epithelial cells have high turnover rates. TFDP1 encodes the transcriptional factor DP-1, which acts as a positive regulator of the G1/S transition during the cell cycle [19], [20]. Particularly in hepatocellular carcinoma, TFDP1 overexpression was substantially associated with disease progression [19]. Since the scope of this current study does not include the functional study of these genes, their biological significance should be investigated in future studies. Recently, Cho *et al.* has performed the largest gene expression profiling in 213 GC patients using fresh frozen tissues [21]. They identified 6 prognostic genes (CTNBB1, EXOCS3, TOP2A, LBA1, CCL5, and LZTR1) for patient survival after curative resection. However, to the best of our knowledge, GCPS is the only gene set which has now been validated in more than 700 stage II GC patients, regardless Lauren classification (diffuse or intestinal), a known prognostic factor, or adjuvant treatment.

In conclusion, with the use of a multistep approach, we developed 8-gene GCPS, which was able to robustly identify high-risk stage II GC patients for recurrence after surgery regardless of adjuvant treatment. Currently, with the ongoing ARTIST-II trial (NCT#01761461), we plan to validate our GCPS in a prospectively designed phase III trial.

## Supporting Information

File S1 **1.** Patient characteristics of study cohorts at each step (**Table S1**). a. Table S1. Patients characteristics. **2.** Clinical and pathological characteristics of cases examined at each phase. **3.** Detailed description of the discovery step using WG-DASL assay (step 1). a. **Figure S1**. QA of WG-DASL data. b. **Table S2**. Comparison of FISH and IHC results for HER2 status in gastric cancer in Step 1. c. **Table S3**. List of probes that are differentially expressed between HER2-positive and HER2- negative patient groups in Step 1. d. **Table S4**. List of all probes with univariate p-values<0.01 in Step 1. e. **Figure S2**. Gradient Lasso algorithm. f. **Table S5**. List of 26 probes included in the prediction model fitted by the whole data set (n = 432). g. **Table S6**. Multivariate Cox regression analysis results in gene discovery set (n = 432). **4.** Design of focused gene expression assay using nCounter platform. a. **Table S7**. List of reference genes for nCounter assay. b. **Figure S3**. Correlation between hazard ratios of prognostic genes based on quantile normalization and self-normalization using WG-DASL assay. **5.** nCounter assay and quality control. **6.** Selection of cut-off for Gastric Cancer Prognostic Score (GCPS(**Figure S4, S5, & S6**). a. **Figure S4**. DFS according to each quartiles of GCPS-g1. b. Figure S5. Cut-point analysis for GCPS-g1. c. **Figure S6**. DFS according to optimized cut-point of GCPS-g1. **6.** Distribution of GCPS between discovery set and validation set. a. **Figure S7**. Distribution of GCPS-g1 within the discovery and validation set. b. **Figure S8**. GCPS: intestinal vs diffuse type. **7.** Testing of clinical utility of GCPS-g2 in patients treated with surgery only. a. **Figure S9**. DFS of stage II patients treated with chemoradiotherapy based on quartile of GCPS-g2. b. **Figure S10**. DFS of stage II patients treated with surgery alone based on quartile of GCPS-g2. c. **Figure S11**. Expression of adverse prognostic genes included in Gastric Cancer Prognostic Score according to tissue compartments (tumor versus stroma). Normalized expression levels are shown. d. **Table S8**. List of nCounter probes included in GCPS-g2. **8.** Gastric cancer validation study protocol. a. **Figure S12**. Gastric cancer validation study protocol.
(DOCX)
