# Supplementary material for: Nanostring-Based Multigene Assay to Predict Recurrence for Gastric Cancer Patients after Surgery
Source: PLoS One. 2014 Mar 5;9(3):e90133. doi: 10.1371/journal.pone.0090133 (PMC3943911; doi:10.1371/journal.pone.0090133)

# **Supporting Information file**

**Nanostring-Based Multigene Assay to Predict Recurrence for Gastric Cancer Patients after Surgery**

**Table of Contents.**

1. Patient characteristics of study cohorts at each step (Table S1)
   1. Table S1. Patients characteristics
2. Clinical and pathological characteristics of cases examined at each phase
3. Detailed description of the discovery step using WG-DASL assay (step 1)
   1. Figure S1. QA of WG-DASL data
   2. Table S2. Comparison of FISH and IHC results for HER2 status in gastric cancer in Step 1
   3. Table S3. List of probes that are differentially expressed between HER2-positive and HER2- negative patient groups in Step 1
   4. Table S4. List of all probes with univariate p-values<0.01 in Step 1
   5. Figure S2. Gradient Lasso algorithm
   6. Table S5. List of 26 probes included in the prediction model fitted by the whole data set (n=432),
   7. Table S6. Multivariate Cox regression analysis results in gene discovery set (n=432)
4. Design of focused gene expression assay using nCounter platform
   1. Table S7. List of reference genes for nCounter assay
   2. Figure S3. Correlation between hazard ratios of prognostic genes based on quantile normalization and self-normalization using WG-DASL assay
5. nCounter assay and quality control
6. Selection of cut-off for Gastric Cancer Prognostic Score (GCPS) (supplementary figure 4, 5, & 6)
   1. Figure S4. DFS according to each quartiles of GCPS-g1
   2. Figure S5. Cut-point analysis for GCPS-g1
   3. Figure S6. DFS according to optimized cut-point of GCPS-g1
7. Distribution of GCPS between discovery set and validation set
   1. Figure S7. Distribution of GCPS-g1 within the discovery and validation set
   2. Figure S8. GCPS: intestinal vs diffuse type
8. Testing of clinical utility of GCPS-g2 in patients treated with surgery only
   1. Figure S9. DFS of stage II patients treated with chemoradiotherapy based on quartile of GCPS-g2
   2. Figure S10. DFS of stage II patients treated with surgery alone based on quartile of GCPS-g2
   3. Figure S11. Expression of adverse prognostic genes included in Gastric Cancer Prognostic Score according to tissue compartments (tumor versus stroma). Normalized expression levels are shown.
   4. Table S8. List of nCounter probes included in GCPS-g2

## 9. Gastric cancer validation study protocol

## a. Figure S12. Gastric cancer validation study protocol 1. Patient population (REMARK Diagram)

From September 1994 to December 2005, from a prospectively collected database maintained at Samsung Medical Center, 1,557 gastric cancer patients were identified who underwent curative gastrectomy and received postoperative adjuvant chemoradiotherapy with 5-fluorouracil plus leucovorin (INT-0116 regimen).

Among those, 1,107 patients were selected based on following criteria: histologically confirmed adenocarcinoma of the stomach; surgical resection of tumor without residual disease, age ≥18, pathology stage IB (T2bN0, T1N1 but not T2aN0) to IV according to the American Joint Committee on Cancer (AJCC) staging system (6th Ed), complete surgical record and treatment record, and patients receiving at least two cycles of INT-0116 regimen. All patients had undergone curative surgical resection and extensive (D2) lymph node dissection with the resection of all perigastric nodes and some celiac, splenic or splenic-hilar, hepatic artery, and cardial lymph nodes, depending on the location of the tumor.

The reasons for case exclusions (N=450) include: inadequate postoperative treatment defined as less than 2 cycles of chemotherapy (N=144), microscopically positive resection margin (N=73), double primary cancer (N=53), recurrent gastric cancer arising from remnant stomach (N=5), insufficient medical record (N=11), use of other than INT-0116 regimen (N=65), insufficient tissue specimen analysis (N=45), and others (N=54). Of the final cohort of 1,107 patients, 520 samples of all clinical stages were randomly selected for the discovery phase of the study and the remaining 587 samples were reserved for validation study.

For phase 4 cohort, among 476 pathologic stage II gastric cancer patients who underwent curative gastrectomy only without adjuvant chemotherapy or post-operation radiotherapy at Samsung Medical Center from 1994 to 2005, 306 patients were selected based on following criteria: histologically confirmed adenocarcinoma of the stomach; surgical resection of tumor without residual disease (R0 gastrectomy); D2 lymph node dissection; age ≥18; pathologic stage II (T1N2, T2aN1, T2bN1 and T3N0); complete surgical record and treatment record. 170 patients will be excluded from the analysis due to the following reasons: insufficient medical record (N=66), death without disease or death with unknown cause (n=43), revised pathologic stage (N=45), no available paraffin blocks (N=15), and double primary cancer (N=1).

## 2. Clinical and pathological characteristics of cases examined at each phase (Table S1).

| **Characteristics** | **Phase 1**  **Microarray (N=432)** | **Phase 2**  **Algorithm development (N=186)** | **Phase 3**  **Algorithm Validation**  **(N=216)** | **Phase 4**  **Test utility in surgery only**  **(N=300)** |
| --- | --- | --- | --- | --- |
| **Age (yr)** |  |  |  |  |
| Median, range | 53, 23 – 74 | 55, 25 – 74 | 53, 23 – 74 |  |
| **Sex** |  |  |  |  |
| Male | 280 (64.8%) | 125 (67.2%) | 155 (71.8%) | 193 (64.3%) |
| Female | 152 (35.2%) | 61 (32.8%) | 61 (28.2%) | 107 (35.7%) |
| **Type of gastrectomy** |  |  |  |  |
| Subtotal gastrectomy | 256 (59.3%) | 124 (66.7%) | 150 (69.4%) | 212 (70.7%) |
| Total gastrectomy | 175 (40.5%) | 61 (32.8%) | 65 (30.1%) | 88 (29.3%) |
| Others | 1 (0.2%) | 1 (0.5%) | 1 (0.5%) | 0 (0.0%) |
| **Location of tumor** |  |  |  |  |
| Distal 1/3 | 231 (53.5%) | 103 (55.4%) | 131 (60.7%) | 183 (61.0%) |
| Middle 1/3 | 130 (30.1%) | 56 (30.1%) | 51 (23.6%) | 85 (28.3%) |
| Cardia, GE junction | 53 (12.3%) | 23 (12.4%) | 24 (11.1%) | 31 (10.3%) |
| Whole, multicentric | 17 (3.9%) | 4 (2.1%) | 10 (4.6%) | 1 (0.3%) |
| Remnant stomach | 1 (0.2%) | 0 (0.0%) | 0 (0.0%) | 0 (0.0%) |
| **Grade** |  |  |  |  |
| W/D~M/D tubular | 111 (25.7%) | 43 (23.1%) | 61 (28.3%) | 128 (42.7%) |
| P/D tubular | 200 (46.3%) | 100 (53.8%) | 101 (46.8%) | 98 (32.7%) |
| Signet ring cell | 101 (23.4%) | 33 (17.7%) | 45 (20.8%) | 60 (20.0%) |
| Mucinous | 14 (3.2%) | 6 (3.2%) | 7 (3.2%) | 8 (2.7%) |
| Papillary | 3 (0.7%) | 2 (1.1%) | 2 (0.9%) | 2 (0.6%) |
| Hepatoid | 2 (0.5%) | 2 (1.1%) | 0 (0.0%) | 2 (0.6%) |
| Others | 1 (0.2%) | 0 (0.0%) | 0 (0.0%) | 2 (0.6%) |
| **Lauren type** |  |  |  |  |
| Intestinal | 139 (32.2%) | 64 (34.4%) | 77 (35.7%) | 174 (58.0%) |
| Diffuse | 280 (64.8%) | 114 (61.3%) | 135 (62.5%) | 122 (40.7%) |
| Mixed | 13 (3.0%) | 8 (4.3%) | 4 (1.8%) | 4 (1.3%) |
| **Lymphovascular invasion** |  |  |  |  |
| Present/identified | 212(49.1%) | 83 (44.6%) | 97 (44.9%) | 223 (74.3%) |
| Not present/Not identified | 220(50.9%) | 103 (55.4%) | 119 (55.1%) | 77 (25.7%) |
| **pT stage** |  |  |  |  |
| T1 | 38(8.8%) | 4 (2.15%) | 10 (4.6%) | 6 (2.0%) |
| T2 | 317 (73.4%) | 178 (95.7%) | 197 (91.2%) | 277 (92.3%) |
| T3 | 77 (17.8%) | 4 (2.15%) | 9 (4.2%) | 17 (5.7%) |
| **pN Stage** |  |  |  |  |
| N0 | 40 (9.3%) | 4 (2.15%) | 9 (4.2%) | 17 (5.7%) |
| N1 | 223 (51.6%) | 178 (95.7%) | 197 (91.2%) | 277 (92.3%) |
| N2 | 103 (23.8%) | 4 (2.15%) | 10 (4.6%) | 6 (2.0%) |
| N3 | 66 (15.3%) | 0 (0.0%) | 0 (0.0%) | 0 (0.0%) |
| **AJCC stage (6^th^ Ed.)** |  |  |  |  |
| Ib | 68 (15.7%) |  |  |  |
| II | 167 (38.7%) | 186 (100%) | 216 (100%) | 300 (100%) |
| IIIA | 111 (25.7%) |  |  |  |
| IIIB | 19 (4.4%) |  |  |  |
| IV | 67 (15.5%) |  |  |  |

**3. Detailed description of the discovery step using WG-DASL assay (step 1)**

We used WG-DASL assay for microarray gene expression profiling of FFPET from the discovery set of 530 patients diagnosed with stage Ib-IV gastric cancer treated with surgery followed by chemoradiotherapy.

A consort diagram describing the study cohort and quality control process is shown in Supplementary Figure 1. Of the final cohort of 1,152 patients, a discovery set of 520 patients were randomly selected and allocated to 6 batches stratified by tumor size and year of surgery for WG-DASL assay. Of 520 samples 27 samples were excluded due to Focus=0 (n=11) and register < 0.75 (n=16) during the scanning of microarray slides. Additional 61 samples were excluded based on following quality control criteria leaving 432 patients for final analyses; low signal (n=21), the number of genes detected (P<0.01) smaller than 4,905 (n=27), and the intensity of the gene corresponding to 75 percentile lower than 500 (n=13).

Out of 24,526 probes, filtering based on absent call in more than 80% of samples resulted in 17,418 probes for subsequent analyses. The intensity of the probes was transformed by logarithm with base 2. Normalization was done using quantile normalization algorithm. Raw WG-DASL data was submitted to GEO database (GSE26253).

**Figure S1. QA of WG-DASL data**


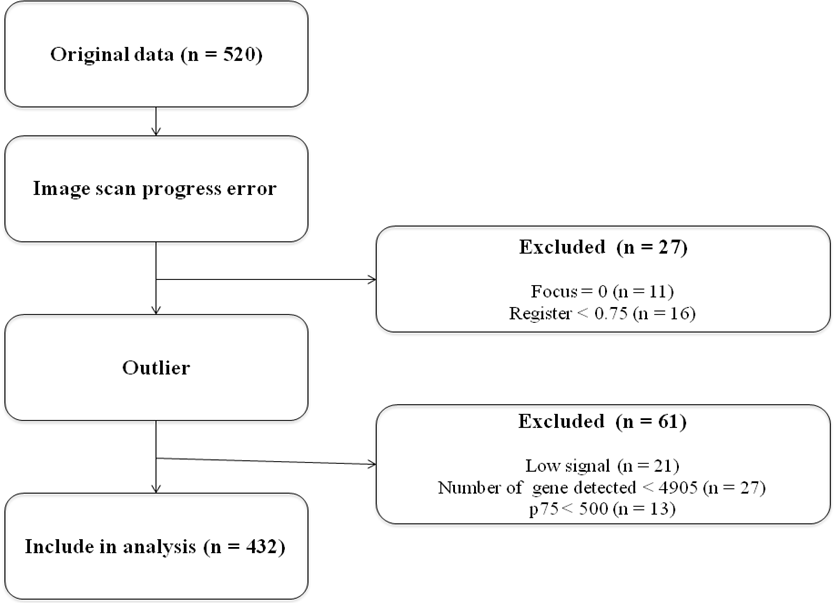


Since WG-DASL assay utilizes fragmented RNA extracted from FFPE blocks, it is important to demonstrate that biologically correct information can be generated using this assay. A subset of gastric cancer has overexpression of HER2 gene located on chromosome 17q as a result of gene amplification. One of the hallmarks of gene amplification is that there are coordinated overexpressions of multiple genes located in the HER2 locus (i.e. GRB7, PERLD1). Hence, for technical validation of WG-DASL assay, we tested whether HER2 gene (ERBB2) or genes physically located close to ERBB2 on chromosome 17 are the main differentially expressed genes between HER2 positive and negative tumors. In order to identify differentially expressed genes between the two groups, we performed t-tests with 100,000 permutations.

HER2 status was determined using both immunohistochemistry (HercepTest) and fluorescence in situ hybridization assay (FISH). For FISH, PathVysion HER2 DNA probe kit (Abbott, Des Plaines, IL) was used. Ariol image analysis system was used to count the hybridization signals (Genetix, San Jose, USA). 40 invasive tumor cells were counted. All samples with ratios of HER2/CEP17 between 1.8 and 2.2 by image analysis were scored manually by counting more than 60 non-overlapping cells. A ratio of more than 2.2, 1.8 to 2.2, or less than 1.8 was classified as positive, equivocal, or negative for amplification, respectively. Chromosomal 17 polysomy was defined as a CEP17 signal more than six copies on average per cell. Table S2 shows the results of HER2 assays for 432 tumors from discovery cohort.

**Table S2.** Comparison of FISH and IHC results for HER2 status in gastric cancer in Step 1

|  | HercepTest^TM^score | | | | Total |
| --- | --- | --- | --- | --- | --- |
|  | 0 | 1+ | 2+ | 3+ |  |
| FISH+ | 9 | 0 | 8 | 36 | 53 |
| FISH- | 280 | 77 | 17 | 0 | 374 |
| FISH-equivocal | 2 | 0 | 3 | 0 | 5 |
| Total | 291 | 77 | 28 | 36 | 432 |

We performed t-tests to identify genes that are differentially expressed between HER2 FISH positive versus negative tumors. We performed 100,000 permutations for t-tests to compensate for non-normal distribution of HER2 status. The results are shown in supplementary table 3. Most of the top differentially expressed genes are from HER2 amplicon including ERBB2 itself demonstrating the analytical validity of WG-DASL platform.

**Table S3.** List of probes that are differentially expressed between HER2-positive and HER2- negative patient groups in Step 1.

| PROBE_ID | SYMBOL | fold change | p-value | q-value | Chromosomal Location |
| --- | --- | --- | --- | --- | --- |
| ILMN_1727078 | C17orf37 | 0.6686 | <0.0001 | 0.0158 | 17q12 |
| ILMN_1728761 | ERBB2 | 1.5039 | <0.0001 | 0.0158 | 17q11.2-q12;17q21.1 |
| ILMN_2352131 | ERBB2 | 1.4116 | <0.0001 | 0.0158 | 17q11.2-q12;17q21.1 |
| ILMN_1740762 | GRB7 | 1.6962 | <0.0001 | 0.0158 | 17q12 |
| ILMN_1798582 | GRB7 | 1.6597 | <0.0001 | 0.0158 | 17q12 |
| ILMN_2405254 | GRB7 | 1.5262 | <0.0001 | 0.0158 | 17q12 |
| ILMN_1666206 | GSDMB | 0.3148 | <0.0001 | 0.0158 | 17q12 |
| ILMN_2366864 | JUP | 0.8402 | <0.0001 | 0.0158 | 17q21 |
| ILMN_1741970 | JUP | 1.4276 | <0.0001 | 0.0158 | 17q21 |
| ILMN_1733811 | JUP | 1.2829 | <0.0001 | 0.0158 | 17q21 |
| ILMN_1666845 | KRT17 | 0.8425 | <0.0001 | 0.0158 | 17q12-q21 |
| ILMN_1805636 | PERLD1 | 1.3792 | <0.0001 | 0.0158 | 17q21 |
| ILMN_2304495 | PPP1R1B | 1.7872 | <0.0001 | 0.0158 | 17q12 |
| ILMN_1748651 | PSMB3 | 0.3227 | <0.0001 | 0.0158 | 17q12 |
| ILMN_1804316 | TCAP | 0.8122 | <0.0001 | 0.0158 | 17q12 |
| ILMN_1686097 | TOP2A | 1.0274 | <0.0001 | 0.0158 | 17q21-q22 |
| ILMN_1791545 | KRT23 | 1.4528 | <0.0001 | 0.0298 | 17q21.2 |
| ILMN_1676215 | DLG2 | -0.6729 | <0.0001 | 0.0422 | 11q14.1 |
| ILMN_1772809 | SLC4A1 | -0.3114 | <0.0001 | 0.0533 | 17q21-q22 |
| ILMN_1665832 | ID1 | 0.8900 | 0.0001 | 0.0633 | 20q11 |
| ILMN_1661708 | LGALS7 | 0.7426 | 0.0001 | 0.0844 | 19q13.2 |
| ILMN_1690096 | PPP1R1B | 1.2019 | 0.0001 | 0.1151 | 17q12 |
| ILMN_1660125 | SFMBT2 | -0.2394 | 0.0001 | 0.1211 | 10p14 |
| ILMN_1714253 | AGRP | -0.1266 | 0.0001 | 0.1372 | 16q22 |
| ILMN_1720501 | ABCA9 | -0.5734 | 0.0001 | 0.1418 | 17q24.2 |
| ILMN_1736056 | DEFB106A | -0.0881 | 0.0002 | 0.1558 | 8p23.1 |
| ILMN_1791366 | RCOR2 | 0.7644 | 0.0002 | 0.1628 | 11q13.1 |
| ILMN_1736078 | THBS4 | -0.6902 | 0.0002 | 0.1628 | 5q13 |
| ILMN_1759012 | PPP1R1B | 1.1659 | 0.0002 | 0.1659 | 17q12 |
| ILMN_1766698 | OR5T1 | -0.0995 | 0.0002 | 0.1941 | 11q12.1 |
| ILMN_1796430 | PSMD3 | 0.7609 | 0.0002 | 0.1960 | 17q21.1 |

Having proven the analytical performance of WG-DASL assay, we searched for prognostic genes using Cox proportional hazard model with gene expression levels treated as continuous variables. Univariate analysis identified 369 probes that were significantly associated with DFS at p<0.01 without adjustment for other clinical prognostic variables. Table S4 lists prognostic genes (p<0.01) from WG-DASL assay.  **Table S4.** List of all probes with univariate p-values<0.01in Step 1

| **PROBE_ID** | **SYMBOL** | **Univariate P-value** | **Hazard ratio** | **Chromosomal Location** |
| --- | --- | --- | --- | --- |
| ILMN_1713561 | C20orf103 | <0.000001 | 1.302187 | 20p12 |
| ILMN_1811790 | FOXS1 | <0.000001 | 1.426582 | 20q11.21 |
| ILMN_1736078 | THBS4 | 0.000001 | 1.320863 | 5q13 |
| ILMN_1672776 | COL10A1 | 0.000001 | 1.657158 | 6q21-q22 |
| ILMN_1732158 | FMO2 | 0.000001 | 1.262049 | 1q23-q25 |
| ILMN_2402392 | COL8A1 | 0.000001 | 1.469684 | 3q12.3 |
| ILMN_2206746 | BGN | 0.000018 | 5.841759 | Xq28 |
| ILMN_1780667 | WDR51A | 0.000024 | 0.727733 | 3p21.2 |
| ILMN_1673843 | CST2 | 0.000026 | 1.289074 | 20p11.21 |
| ILMN_1775931 | EPHA3 | 0.000032 | 1.313913 | 3p11.2 |
| ILMN_1749846 | OMD | 0.000033 | 1.360159 | 9q22.31 |
| ILMN_1755318 | HIST1H2AJ | 0.000036 | 0.660616 | 6p22-p21.3 |
| ILMN_1677636 | COMP | 0.000043 | 1.204699 | 19p13.1 |
| ILMN_2316386 | GPBAR1 | 0.000049 | 2.746716 | 2q35 |
| ILMN_1740265 | ACOT7 | 0.000056 | 0.487482 | 1p36.31-p36.11 |
| ILMN_1774350 | MYOZ3 | 0.000077 | 1.2456 | 5q33.1 |
| ILMN_2093500 | ZBED5 | 0.000081 | 1.403335 | 11p15.3 |
| ILMN_1701331 | UBE2M | 0.000084 | 0.139592 | 19q13.43 |
| ILMN_2071809 | MGP | 0.000100 | 1.89168 | 12p13.1-p12.3 |
| ILMN_1759792 | CLIP4 | 0.000103 | 1.264165 | 2p23.2 |
| ILMN_2188451 | HIST1H2AH | 0.000110 | 0.648883 | 6p21.33 |
| ILMN_2138589 | MERTK | 0.000117 | 1.344156 | 2q14.1 |
| ILMN_1735996 | NOX4 | 0.000130 | 1.273066 | 11q14.2-q21 |
| ILMN_1782329 | HIST1H4L | 0.000131 | 0.752221 | 6p22-p21.3 |
| ILMN_1726603 | ATP5I | 0.000137 | 0.076217 | 4p16.3 |
| ILMN_1695079 | ZNF101 | 0.000146 | 0.669062 | 19p13.11 |
| ILMN_1797693 | BRI3BP | 0.000171 | 0.410778 | 12q24.31 |
| ILMN_1653553 | C14orf80 | 0.000190 | 0.500153 | 14q32.33 |
| ILMN_1792538 | CD7 | 0.000192 | 0.43232 | 17q25.2-q25.3 |
| ILMN_1757387 | UCHL1 | 0.000201 | 1.653798 | 4p14 |
| ILMN_1693597 | ZNF287 | 0.000208 | 1.194469 | 17p11.2 |
| ILMN_1673548 | HSPC159 | 0.000209 | 0.680392 | 2p14 |
| ILMN_1753524 | HIST1H2AB | 0.000211 | 0.72192 | 6p21.3 |
| ILMN_2382679 | REG3A | 0.000232 | 0.863324 | 2p12 |
| ILMN_1769168 | ARL10 | 0.000235 | 1.225049 | 5q35.2 |
| ILMN_2071826 | RNF152 | 0.000264 | 1.232257 | 18q21.33 |
| ILMN_1719543 | MAF | 0.000267 | 1.166273 | 16q22-q23 |
| ILMN_1711566 | TIMP1 | 0.000268 | 5.209361 | Xp11.3-p11.23 |
| ILMN_2163873 | FNDC1 | 0.000292 | 1.275913 | 6q25 |
| ILMN_1685433 | COL8A1 | 0.000300 | 1.530905 | 3q12.3 |
| ILMN_2115696 | USP42 | 0.000308 | 1.158447 | 7p22.1 |
| ILMN_1801205 | GPNMB | 0.000313 | 1.298661 | 7p15 |
| ILMN_1712430 | ATP5G1 | 0.000346 | 0.76876 | 17q21.32 |
| ILMN_1710752 | NAPRT1 | 0.000351 | 0.281588 | 8q24.3 |
| ILMN_2168166 | ASPN | 0.000355 | 1.390402 | 9q22 |
| ILMN_1787749 | CASP8 | 0.000365 | 0.779592 | 2q33-q34 |
| ILMN_1727709 | GPBAR1 | 0.000365 | 1.285664 | 2q35 |
| ILMN_1765557 | OLFML2B | 0.000368 | 1.226139 | 1q23.3 |
| ILMN_1796734 | SPARC | 0.000397 | 1.216715 | 5q31.3-q32 |
| ILMN_2392803 | COL11A1 | 0.000398 | 1.203319 | 1p21 |
| ILMN_1750180 | HIST1H2BB | 0.000405 | 0.762857 | 6p21.3 |
| ILMN_2300970 | ETFB | 0.000405 | 0.26307 | 19q13.3 |
| ILMN_2396875 | IGFBP3 | 0.000413 | 1.252929 | 7p13-p12 |
| ILMN_1750052 | NOL14 | 0.000417 | 0.519811 | 4p16.3 |
| ILMN_2151368 | NOL12 | 0.000430 | 1.217182 | 22q13.1 |
| ILMN_2330570 | LEPR | 0.000455 | 1.303249 | 1p31 |
| ILMN_1732782 | SCN2A | 0.000467 | 1.210705 | 2q23-q24 |
| ILMN_1708143 | FAM127A | 0.000473 | 1.250246 | Xq26 |
| ILMN_2219867 | KRT20 | 0.000544 | 0.761421 | 17q21.2 |
| ILMN_1800331 | PTCH1 | 0.000571 | 1.248174 | 9q22.3 |
| ILMN_1726815 | HIST1H3G | 0.000597 | 0.777786 | 6p21.3 |
| ILMN_1677652 | PREX2 | 0.000601 | 1.168492 | 8q13.2 |
| ILMN_1758067 | RGS4 | 0.000603 | 1.239717 | 1q23.3 |
| ILMN_1712751 | HADHA | 0.000650 | 0.260972 | 2p23 |
| ILMN_1710522 | RUNX1T1 | 0.000655 | 1.238393 | 8q22 |
| ILMN_1712532 | CARD9 | 0.000658 | 1.29096 | 9q34.3 |
| ILMN_1770290 | CNN2 | 0.000690 | 3.212723 | 19p13.3 |
| ILMN_2355786 | BTNL3 | 0.000751 | 0.704169 | 5q35.3 |
| ILMN_1749878 | FAM124B | 0.000755 | 1.269953 | 2q36.2 |
| ILMN_1712088 | CLYBL | 0.000780 | 0.804096 | 13q32 |
| ILMN_1677747 | TMPO | 0.000783 | 0.716288 | 12q22 |
| ILMN_1675706 | APOA4 | 0.000795 | 0.83371 | 11q23 |
| ILMN_2073184 | S1PR5 | 0.000801 | 1.213767 | 19p13.2 |
| ILMN_1735877 | EFEMP1 | 0.000844 | 1.289706 | 2p16 |
| ILMN_1758128 | CYGB | 0.000856 | 1.926103 | 17q25.3 |
| ILMN_1729033 | RPL9 | 0.000901 | 0.826367 | 4p13 |
| ILMN_1678669 | RRM2 | 0.000917 | 0.848107 | 2p25-p24 |
| ILMN_1809866 | WDR74 | 0.000925 | 0.479147 | 11q12.3 |
| ILMN_1662824 | MADCAM1 | 0.000956 | 0.774827 | 19p13.3 |
| ILMN_2328094 | DACT1 | 0.000977 | 1.243186 | 14q23.1 |
| ILMN_1729368 | FZD8 | 0.001017 | 1.195987 | 10p11.21 |
| ILMN_1738116 | TMEM119 | 0.001036 | 1.182145 | 12q23.3 |
| ILMN_1810486 | RAB34 | 0.001053 | 1.220139 | 17q11.2 |
| ILMN_1712400 | SERPINB6 | 0.001071 | 0.214418 | 6p25 |
| ILMN_1780170 | APOD | 0.001092 | 1.201164 | 3q26.2-qter |
| ILMN_1671557 | PHLDA2 | 0.001116 | 0.736297 | 11p15.5 |
| ILMN_2077094 | C11orf2 | 0.001117 | 0.405725 | 11q13 |
| ILMN_2219681 | RBP2 | 0.001131 | 0.741382 | 3q23 |
| ILMN_2051972 | GPC3 | 0.001168 | 1.214438 | Xq26.1 |
| ILMN_2372200 | ZNF586 | 0.001199 | 0.568325 | 19q13.43 |
| ILMN_1738684 | NRXN2 | 0.001203 | 1.202815 | 11q13 |
| ILMN_1792748 | CPS1 | 0.001224 | 0.827387 | 2q35 |
| ILMN_1752299 | RAB6B | 0.001235 | 1.190276 | 3q22.1 |
| ILMN_1701403 | HIP1 | 0.001245 | 1.219317 | 7q11.23 |
| ILMN_1763491 | CKMT1B | 0.001280 | 0.80908 | 15q15 |
| ILMN_1722898 | SFRP2 | 0.001291 | 1.269536 | 4q31.3 |
| ILMN_1705468 | PIK3CA | 0.001310 | 1.226938 | 3q26.3 |
| ILMN_1803570 | BRI3BP | 0.001319 | 0.867957 | 12q24.31 |
| ILMN_1776077 | SF1 | 0.001347 | 1.309907 | 11q13 |
| ILMN_2118129 | ITLN2 | 0.001364 | 0.872461 | 1q22-q23 |
| ILMN_1717163 | F13A1 | 0.001373 | 1.300682 | 6p25.3-p24.3 |
| ILMN_1692739 | ISLR2 | 0.001375 | 1.196592 | 15q24.1 |
| ILMN_2067709 | TFB2M | 0.001385 | 0.653243 | 1q44 |
| ILMN_1697363 | C20orf27 | 0.001432 | 0.714003 | 20p13 |
| ILMN_1740523 | KTN1 | 0.001500 | 1.203121 | 14q22.1 |
| ILMN_2398664 | RNF34 | 0.001501 | 0.504034 | 12q24.31 |
| ILMN_2148469 | RASL11B | 0.001508 | 1.149716 | 4q12 |
| ILMN_1747067 | NPAS1 | 0.001542 | 0.781286 | 19q13.2-q13.3 |
| ILMN_1792110 | C10orf76 | 0.001576 | 1.184364 | 10q24.32 |
| ILMN_1714438 | MUTYH | 0.001606 | 1.246579 | 1p34.3-p32.1 |
| ILMN_1651964 | ABCC5 | 0.001622 | 1.21205 | 3q27 |
| ILMN_1764709 | MAFB | 0.001623 | 1.207708 | 20q11.2-q13.1 |
| ILMN_1652461 | PARD3B | 0.001645 | 1.255893 | 2q33.3 |
| ILMN_1694539 | MAP3K6 | 0.001647 | 1.197227 | 1p36.11 |
| ILMN_1765532 | RDBP | 0.001667 | 0.463459 | 6p21.3 |
| ILMN_1711009 | ISLR | 0.001668 | 1.196724 | 15q23-q24 |
| ILMN_1811426 | TMTC1 | 0.001677 | 1.226595 | 12p11.22 |
| ILMN_1702806 | PDCL3 | 0.001685 | 0.589571 | 2q11.2 |
| ILMN_1688067 | SEPT5 | 0.001746 | 1.142858 | 22q11.21 |
| ILMN_1770800 | PODN | 0.001767 | 2.010032 | 1p32.3 |
| ILMN_1787115 | WWTR1 | 0.001836 | 1.205927 | 3q23-q24 |
| ILMN_1757060 | CAMK2D | 0.001909 | 0.658325 | 4q26 |
| ILMN_2350634 | EFEMP1 | 0.001942 | 1.276171 | 2p16 |
| ILMN_1672611 | CDH11 | 0.001960 | 1.235423 | 16q22.1 |
| ILMN_2234310 | GLTPD1 | 0.001975 | 1.183448 | 1p36.33 |
| ILMN_1680419 | ASB7 | 0.001983 | 0.664755 | 15q26.3 |
| ILMN_1673586 | SLC6A6 | 0.002020 | 1.368342 | 3p25-p24 |
| ILMN_1777263 | MEOX2 | 0.002037 | 1.200134 | 7p22.1-p21.3 |
| ILMN_1670903 | NAT2 | 0.002038 | 0.678685 | 8p22 |
| ILMN_1673721 | EXO1 | 0.002053 | 0.692379 | 1q42-q43 |
| ILMN_1667112 | FBXO7 | 0.002055 | 0.716782 | 22q12-q13 |
| ILMN_2273911 | ACSL5 | 0.002090 | 0.784848 | 10q25.1-q25.2 |
| ILMN_1734950 | LOXL1 | 0.002152 | 1.22384 | 15q24-q25;15q22 |
| ILMN_1793965 | PCDHGA8 | 0.002196 | 0.767728 | 5q31 |
| ILMN_1733396 | CDC25A | 0.002317 | 0.837309 | 3p21 |
| ILMN_1729188 | HAMP | 0.002365 | 1.168372 | 19q13.1 |
| ILMN_1718646 | MMP15 | 0.002444 | 0.85565 | 16q13-q21 |
| ILMN_2230178 | DAND5 | 0.002497 | 0.764252 | 19p13.2 |
| ILMN_1767665 | LOC493869 | 0.002565 | 1.187629 | 5q11.2 |
| ILMN_1657683 | C1orf198 | 0.002578 | 1.288479 | 1q42.13-q43 |
| ILMN_1741356 | PRICKLE1 | 0.002616 | 1.505195 | 12q12 |
| ILMN_1677043 | AKR7A2 | 0.002709 | 0.468292 | 1p36.13 |
| ILMN_1721559 | FABP6 | 0.002889 | 1.130136 | 5q33.3-q34 |
| ILMN_1720496 | GUCY1A2 | 0.002901 | 1.22875 | 11q21-q22 |
| ILMN_2159044 | PDF | 0.002908 | 0.676684 | 16q22.1 |
| ILMN_1708369 | EPS15L1 | 0.002965 | 1.214545 | 19p13.11 |
| ILMN_1743579 | WDR4 | 0.002985 | 0.73777 | 21q22.3 |
| ILMN_2249018 | LOC389816 | 0.003016 | 0.773289 | 9q34.3 |
| ILMN_1798379 | HNT | 0.003065 | 1.19188 | 11q25 |
| ILMN_1779558 | GAS6 | 0.003092 | 1.4833 | 13q34 |
| ILMN_2150095 | CES4 | 0.003155 | 1.161699 | 16q12.2 |
| ILMN_1726392 | NIN | 0.003165 | 0.684082 | 14q22.1 |
| ILMN_2044832 | NOL5A | 0.003244 | 0.750471 | 20p13 |
| ILMN_1742238 | SET | 0.003279 | 0.688373 | 9q34 |
| ILMN_1662523 | C3 | 0.003297 | 1.660256 | 19p13.3-p13.2 |
| ILMN_1778924 | PDE1A | 0.003326 | 1.172318 | 2q32.1 |
| ILMN_2314140 | PAX6 | 0.003344 | 0.616935 | 11p13 |
| ILMN_1737817 | CCL25 | 0.003382 | 0.894426 | 19p13.2 |
| ILMN_2381296 | GSTZ1 | 0.003384 | 0.755576 | 14q24.3 |
| ILMN_1714041 | PLCB3 | 0.003396 | 0.730519 | 11q13 |
| ILMN_1668714 | SNF1LK2 | 0.003424 | 1.17226 | 11q23.1 |
| ILMN_1743103 | SH3PXD2A | 0.003465 | 1.16748 | 10q24.33 |
| ILMN_1755737 | TRABD | 0.003492 | 0.259653 | 22q13.33 |
| ILMN_1795228 | ZFAND5 | 0.003506 | 0.824405 | 9q13-q21 |
| ILMN_1654737 | TRIM32 | 0.003522 | 0.79033 | 9q33.1 |
| ILMN_1752249 | FAM38A | 0.003530 | 2.322901 | 16q24.3 |
| ILMN_1730740 | VSIG8 | 0.003570 | 1.197703 | 1q23.2 |
| ILMN_1672660 | MBP | 0.003626 | 1.145594 | 18q23 |
| ILMN_1738883 | RNF135 | 0.003632 | 0.631082 | 17q11.2 |
| ILMN_1718387 | LOR | 0.003648 | 1.140323 | 1q21 |
| ILMN_1718754 | CD207 | 0.003649 | 1.139594 | 2p13 |
| ILMN_2392261 | FABP6 | 0.003697 | 1.128338 | 5q33.3-q34 |
| ILMN_1653251 | HIST1H1B | 0.003721 | 0.759297 | 6p22-p21.3 |
| ILMN_1736178 | AEBP1 | 0.003766 | 1.540385 | 7p13 |
| ILMN_2253732 | ST8SIA4 | 0.003779 | 1.242747 | 5q21 |
| ILMN_1812795 | RUNX1T1 | 0.003809 | 1.130963 | 8q22 |
| ILMN_1779373 | HIST1H2BF | 0.003828 | 0.82018 | 6p21.3 |
| ILMN_1753823 | IL17D | 0.003852 | 1.156443 | 13q12.11 |
| ILMN_1676311 | COX18 | 0.003858 | 0.70773 | 4q13.3 |
| ILMN_1809267 | CLCC1 | 0.003864 | 1.167778 | 1p13.3 |
| ILMN_1723035 | OLR1 | 0.003871 | 1.188039 | 12p13.2-p12.3 |
| ILMN_1720838 | DECR1 | 0.003908 | 0.644174 | 8q21.3 |
| ILMN_1791569 | PLXNA1 | 0.003943 | 1.230557 | 3q21.3 |
| ILMN_2223056 | TBX10 | 0.003953 | 0.78329 | 11q13.2 |
| ILMN_1761084 | FNDC5 | 0.004021 | 1.255781 | 1p35.1 |
| ILMN_2323338 | NR1I2 | 0.004034 | 0.72455 | 3q12-q13.3 |
| ILMN_2340131 | MAPK10 | 0.004035 | 1.186702 | 4q22.1-q23 |
| ILMN_2062468 | IGFBP7 | 0.004039 | 1.445932 | 4q12 |
| ILMN_1653940 | USP2 | 0.004084 | 0.705592 | 11q23.3 |
| ILMN_2276461 | MAP2 | 0.004109 | 1.185406 | 2q34-q35 |
| ILMN_2215881 | ARHGAP11B | 0.004154 | 0.844611 | 15q13.2 |
| ILMN_1678170 | MME | 0.004174 | 0.838642 | 3q25.1-q25.2 |
| ILMN_2186983 | ANXA8L2 | 0.004193 | 1.118791 | 10q11.22 |
| ILMN_1758209 | UACA | 0.004224 | 1.240201 | 15q22-q24 |
| ILMN_1663171 | MATN3 | 0.004238 | 1.341007 | 2p24-p23 |
| ILMN_1749789 | HIST1H1D | 0.004263 | 0.6848 | 6p21.3 |
| ILMN_1696675 | CES2 | 0.004291 | 0.557116 | 16q22.1 |
| ILMN_1721127 | HIST1H3D | 0.004306 | 0.697261 | 6p21.3 |
| ILMN_1680314 | TXN | 0.004463 | 0.832985 | 9q31 |
| ILMN_1790026 | SFRP5 | 0.004465 | 1.120384 | 10q24.1 |
| ILMN_2103685 | DEPDC1B | 0.004470 | 0.706872 | 5q12.1 |
| ILMN_1723111 | HIST1H4A | 0.004505 | 0.844986 | 6p21.3 |
| ILMN_2212878 | ESM1 | 0.004516 | 1.298296 | 5q11.2 |
| ILMN_1654946 | ZSCAN18 | 0.004528 | 1.147692 | 19q13.43 |
| ILMN_1661010 | ZMAT1 | 0.004591 | 1.147018 | Xq21 |
| ILMN_1813625 | TRIM25 | 0.004620 | 0.825171 | 17q23.2 |
| ILMN_1791006 | AHI1 | 0.004662 | 1.181987 | 6q23.3 |
| ILMN_1660079 | RNF44 | 0.004707 | 1.349436 | 5q35.2 |
| ILMN_2095660 | TMEM156 | 0.004727 | 0.677499 | 4p14 |
| ILMN_1687652 | TGFB3 | 0.004840 | 1.158037 | 14q24 |
| ILMN_1765189 | PTK2B | 0.004858 | 0.709154 | 8p21.1 |
| ILMN_1654920 | HNRPH3 | 0.004879 | 1.226349 | 10q22 |
| ILMN_1678710 | PHYHIPL | 0.004902 | 1.296577 | 10q11 |
| ILMN_1748591 | ODC1 | 0.004913 | 0.843751 | 2p25 |
| ILMN_2413278 | RPL13 | 0.004966 | 0.633774 | 16q24.3;17p11.2 |
| ILMN_2354855 | OTUB1 | 0.005013 | 0.456697 | 11q13.1 |
| ILMN_1747146 | TSG101 | 0.005020 | 0.740316 | 11p15 |
| ILMN_1792682 | MCTP2 | 0.005074 | 1.214269 | 15q26.2 |
| ILMN_2401779 | FAM102A | 0.005094 | 0.480071 | 9q34.11 |
| ILMN_1667641 | ACACA | 0.005104 | 1.164908 | 17q21 |
| ILMN_2106818 | MBIP | 0.005113 | 1.299724 | 14q13.3 |
| ILMN_2324989 | IKIP | 0.005168 | 0.825236 | 12q23.1 |
| ILMN_1741994 | L3MBTL3 | 0.005177 | 1.15951 | 6q23 |
| ILMN_2223941 | FBLN5 | 0.005178 | 1.411779 | 14q32.1 |
| ILMN_2092536 | HSPE1 | 0.005197 | 0.635259 | 2q33.1 |
| ILMN_1752226 | P2RY11 | 0.005209 | 0.481078 | 19p13.2 |
| ILMN_1784871 | FASN | 0.005254 | 0.291412 | 17q25 |
| ILMN_1677385 | C8orf40 | 0.005319 | 1.225286 | 8p11.21 |
| ILMN_1736112 | ARHGAP10 | 0.005348 | 1.242786 | 4q31.23 |
| ILMN_2229649 | KCTD12 | 0.005351 | 1.219597 | 13q22.3 |
| ILMN_1669497 | OSBPL10 | 0.005372 | 1.17703 | 3p22.3 |
| ILMN_1665260 | FLJ25996 | 0.005385 | 1.214995 | 3q29 |
| ILMN_2355033 | KIAA1147 | 0.005454 | 1.203143 | 7q34 |
| ILMN_1768393 | SNRPD1 | 0.005693 | 0.702623 | 18q11.2 |
| ILMN_1671058 | CDX2 | 0.005696 | 0.857541 | 13q12.3 |
| ILMN_1704730 | CD93 | 0.005717 | 1.153984 | 20p11.21 |
| ILMN_1717888 | KHK | 0.005740 | 0.833244 | 2p23.3 |
| ILMN_2385647 | ALAS1 | 0.005756 | 0.857815 | 3p21.1 |
| ILMN_2385672 | ELN | 0.005759 | 1.253974 | 7q11.23 |
| ILMN_1754655 | TTLL5 | 0.005810 | 0.810275 | 14q24.3 |
| ILMN_1711005 | CDC25A | 0.005869 | 0.770725 | 3p21 |
| ILMN_2186137 | RRAD | 0.005904 | 1.289988 | 16q22 |
| ILMN_1769782 | LAX1 | 0.005938 | 0.838368 | 1q32.1 |
| ILMN_1738552 | SLC1A3 | 0.005942 | 1.301663 | 5p13 |
| ILMN_1726204 | SCRG1 | 0.005979 | 1.165889 | 4q31-q32 |
| ILMN_2390526 | RARB | 0.005985 | 1.386201 | 3p24 |
| ILMN_1695631 | CHP2 | 0.005994 | 0.827999 | 16p12.2 |
| ILMN_1786612 | PSME2 | 0.006016 | 0.649769 | 14q11.2 |
| ILMN_1747716 | ALDOB | 0.006038 | 0.890305 | 9q21.3-q22.2 |
| ILMN_2234187 | CDO1 | 0.006053 | 1.25975 | 5q22-q23 |
| ILMN_1761000 | ASAH3L | 0.006076 | 0.85266 | 9p22.1 |
| ILMN_2121272 | PDE10A | 0.006077 | 1.164521 | 6q26 |
| ILMN_1813295 | LMO3 | 0.006127 | 1.200168 | 12p12.3 |
| ILMN_1686804 | CCRK | 0.006219 | 1.2044 | 9q22.1 |
| ILMN_1736176 | PLK1 | 0.006220 | 0.779772 | 16p12.2 |
| ILMN_1779448 | EFHD1 | 0.006236 | 1.163807 | 2q37.1 |
| ILMN_1788729 | TCF23 | 0.006252 | 1.172746 | 2p23.3 |
| ILMN_1657836 | PLEKHG2 | 0.006326 | 1.256047 | 19q13.2 |
| ILMN_1758597 | NAGS | 0.006357 | 0.826079 | 17q21.31 |
| ILMN_1731374 | CPE | 0.006365 | 1.172434 | 4q32.3 |
| ILMN_2125395 | GPR128 | 0.006386 | 0.860916 | 3q12.2 |
| ILMN_1670638 | PITPNC1 | 0.006404 | 1.174446 | 17q24.2 |
| ILMN_1658989 | MEX3B | 0.006405 | 0.660644 | 15q25.2 |
| ILMN_1712065 | FAM19A5 | 0.006442 | 1.132281 | 22q13.32 |
| ILMN_1717261 | HLA-DRB3 | 0.006482 | 0.877062 | 6p21.3 |
| ILMN_1663786 | EPB41 | 0.006503 | 0.757261 | 1p33-p32 |
| ILMN_1692511 | TMEM106C | 0.006510 | 0.630838 | 12q13.1 |
| ILMN_1654246 | SIRT6 | 0.006513 | 0.864446 | 19p13.3 |
| ILMN_1811278 | RNF186 | 0.006532 | 0.704422 | 1p36.13 |
| ILMN_1700306 | OCIAD2 | 0.006614 | 0.845453 | 4p11 |
| ILMN_2141444 | RPL18A | 0.006624 | 0.140945 | 19p13 |
| ILMN_1754576 | KRT6C | 0.006692 | 1.137425 | 12q13.13 |
| ILMN_1745329 | PRR14 | 0.006695 | 0.65701 | 16p11.2 |
| ILMN_1772645 | AGK | 0.006759 | 1.207978 | 7q34 |
| ILMN_1766425 | REPS2 | 0.006798 | 1.224906 | Xp22.2-p22.13 |
| ILMN_1803376 | AEBP2 | 0.006913 | 0.688622 | 12p12.3 |
| ILMN_1695093 | SLC7A8 | 0.006950 | 0.803152 | 14q11.2 |
| ILMN_1719089 | EXO1 | 0.006962 | 0.746364 | 1q42-q43 |
| ILMN_1665832 | ID1 | 0.006984 | 0.853496 | 20q11 |
| ILMN_1675219 | WDHD1 | 0.007008 | 0.790273 | 14q22.2 |
| ILMN_1797219 | CLCA1 | 0.007035 | 0.877425 | 1p31-p22 |
| ILMN_1739594 | ACOT11 | 0.007105 | 0.637043 | 1p32.3 |
| ILMN_2094942 | MARCH1 | 0.007129 | 1.183848 | 4q32.2 |
| ILMN_1657495 | KIAA0152 | 0.007174 | 0.714483 | 12q24.31 |
| ILMN_1729287 | NMUR1 | 0.007175 | 1.19547 | 2q37.1 |
| ILMN_1804090 | SLC25A10 | 0.007205 | 0.697334 | 17q25.3 |
| ILMN_1713807 | MAN1C1 | 0.007212 | 1.218037 | 1p35 |
| ILMN_1801068 | DACT1 | 0.007228 | 1.155005 | 14q23.1 |
| ILMN_2316236 | HOPX | 0.007247 | 1.239065 | 4q11-q12 |
| ILMN_1736670 | PPP1R3C | 0.007302 | 1.2415 | 10q23-q24 |
| ILMN_1676058 | MAGOHB | 0.007329 | 0.706763 | 12p13.2 |
| ILMN_1813207 | MRPS9 | 0.007392 | 0.85342 | 2q12.1 |
| ILMN_2389935 | FYTTD1 | 0.007408 | 0.707889 | 3q29 |
| ILMN_2387995 | ANTXR1 | 0.007426 | 1.291281 | 2p13.1 |
| ILMN_1740160 | PLCG1 | 0.007472 | 1.372496 | 20q12-q13.1 |
| ILMN_2299862 | KCNH1 | 0.007479 | 0.732409 | 1q32-q41 |
| ILMN_1715401 | MT1G | 0.007518 | 0.815893 | 16q13 |
| ILMN_2232854 | FAP | 0.007528 | 1.122417 | 2q23 |
| ILMN_1712506 | DPP6 | 0.007555 | 0.808478 | 7q36.2 |
| ILMN_2289623 | TTC36 | 0.007579 | 0.847973 | 11q23.3 |
| ILMN_2241168 | MAFF | 0.007592 | 0.851913 | 22q13.1 |
| ILMN_1665761 | BCL11B | 0.007679 | 0.856148 | 14q32.2 |
| ILMN_1721495 | ADAMTSL2 | 0.007681 | 1.468854 | 9q34.2 |
| ILMN_1811277 | TRIM13 | 0.007696 | 0.782911 | 13q14 |
| ILMN_1719616 | DNASE1 | 0.007756 | 0.799984 | 16p13.3 |
| ILMN_1744387 | KCNIP1 | 0.007790 | 1.185425 | 5q35.1 |
| ILMN_1810274 | HOXB2 | 0.007796 | 1.183641 | 17q21-q22 |
| ILMN_1776490 | C17orf53 | 0.007804 | 0.797121 | 17q21.31 |
| ILMN_1776314 | CHRNA10 | 0.007829 | 0.753693 | 11p15.5 |
| ILMN_2398184 | NCAM1 | 0.007903 | 0.721315 | 11q23.1 |
| ILMN_2405592 | TMEM93 | 0.007932 | 0.816861 | 17p13.2 |
| ILMN_1661695 | IRAK3 | 0.007934 | 1.16293 | 12q14.3 |
| ILMN_1758852 | ENTPD7 | 0.007942 | 0.786463 | 10q23 |
| ILMN_1784749 | GAS6 | 0.007988 | 1.496952 | 13q34 |
| ILMN_1664828 | APOBEC3H | 0.008019 | 1.149546 | 22q13.1 |
| ILMN_1735827 | NISCH | 0.008030 | 1.848072 | 3p21.1 |
| ILMN_2072296 | CKS2 | 0.008106 | 0.875387 | 9q22 |
| ILMN_1683905 | C19orf21 | 0.008128 | 0.674629 | 19p13.3 |
| ILMN_1813206 | CP | 0.008182 | 1.139449 | 3q23-q25 |
| ILMN_1774742 | MTTP | 0.008191 | 0.851238 | 4q24 |
| ILMN_1745108 | ADAD2 | 0.008272 | 0.79017 | 16q24.1 |
| ILMN_1805404 | GRIN1 | 0.008324 | 0.825892 | 9q34.3 |
| ILMN_2341006 | SCARF2 | 0.008346 | 1.146775 | 22q11.21 |
| ILMN_1711766 | SKP1A | 0.008391 | 1.167702 | 5q31 |
| ILMN_1773080 | OAZ1 | 0.008426 | 0.115385 | 19p13.3 |
| ILMN_1673069 | DPP9 | 0.008544 | 0.866548 | 19p13.3 |
| ILMN_2362368 | U2AF1 | 0.008629 | 0.733454 | 21q22.3 |
| ILMN_1769092 | FAM176B | 0.008661 | 1.123628 | 1p34.3 |
| ILMN_1724754 | MPP3 | 0.008709 | 0.782568 | 17q21.31 |
| ILMN_1750981 | SLC25A26 | 0.008735 | 0.826359 | 3p14.1 |
| ILMN_2160005 | NUMA1 | 0.008776 | 1.173853 | 11q13 |
| ILMN_1654324 | HEYL | 0.008779 | 1.439102 | 1p34.3 |
| ILMN_1759184 | C19orf48 | 0.008783 | 0.491992 | 19q13.33 |
| ILMN_1815556 | PRAP1 | 0.008801 | 0.865457 | 10q26.3 |
| ILMN_2112417 | PGAM1 | 0.008809 | 0.739931 | 10q25.3 |
| ILMN_1718265 | ATG5 | 0.008841 | 0.647927 | 6q21 |
| ILMN_1697812 | HLXB9 | 0.008859 | 0.80677 | 7q36 |
| ILMN_1720300 | PRR5 | 0.008942 | 0.699181 | 22q13 |
| ILMN_1806432 | NT5C | 0.009019 | 0.523857 | 17q25.1 |
| ILMN_1736154 | ProSAPiP1 | 0.009019 | 1.138612 | 20p13 |
| ILMN_1726786 | TNRC6B | 0.009044 | 1.146365 | 22q13.1 |
| ILMN_1682226 | CLDN15 | 0.009051 | 0.861745 | 7q11.22 |
| ILMN_2242900 | IL1RL1 | 0.009160 | 0.809737 | 2q12 |
| ILMN_2343618 | SAMD3 | 0.009198 | 1.149687 | 6q23.1 |
| ILMN_1707513 | PGPEP1 | 0.009230 | 0.804627 | 19p13.11 |
| ILMN_1734766 | C6orf182 | 0.009234 | 0.701394 | 6q21 |
| ILMN_1702363 | SULF1 | 0.009314 | 1.180017 | 8q13.2-q13.3 |
| ILMN_1693250 | ACBD5 | 0.009316 | 0.728409 | 10p12.1 |
| ILMN_2322375 | MAFF | 0.009335 | 0.724285 | 22q13.1 |
| ILMN_1720114 | GMNN | 0.009355 | 0.791881 | 6p22.3 |
| ILMN_1753789 | TNN | 0.009396 | 1.141063 | 1q23-q24 |
| ILMN_2108493 | TMEM120B | 0.009404 | 1.167851 | 12q24.31 |
| ILMN_1735594 | CDC42SE2 | 0.009429 | 0.764487 | 5q23.3-q31.1 |
| ILMN_2351230 | RUFY3 | 0.009442 | 1.169369 | 4q13.3 |
| ILMN_1662438 | SOD1 | 0.009447 | 0.504471 | 21q22.1;21q22.11 |
| ILMN_2116556 | LSM5 | 0.009490 | 0.66213 | 7p14.3 |
| ILMN_1787691 | CITED4 | 0.009495 | 1.144128 | 1p34.2;1p35-p34 |
| ILMN_1706579 | SHBG | 0.009582 | 0.841448 | 17p13-p12 |
| ILMN_2388517 | MTERFD3 | 0.009691 | 0.664478 | 12q24.1 |
| ILMN_1800590 | BBS1 | 0.009711 | 1.143601 | 11q13.1 |
| ILMN_1709044 | TGIF2 | 0.009722 | 0.750476 | 20q11.2-q12 |
| ILMN_1803956 | BOC | 0.009754 | 1.22073 | 3q13.2 |
| ILMN_1730734 | TMEM205 | 0.009780 | 0.379033 | 19p13.2 |
| ILMN_2330787 | FRMD6 | 0.009801 | 1.243165 | 14q22.1 |
| ILMN_1661875 | ANK3 | 0.009868 | 0.814233 | 10q21 |
| ILMN_1748077 | DDX59 | 0.009902 | 1.232856 | 1q32.1 |
| ILMN_2343036 | ZMYM5 | 0.009915 | 1.154223 | 13q12 |
| ILMN_2388669 | GRIA3 | 0.009940 | 0.778696 | Xq25-q26 |
| ILMN_1748283 | PIM2 | 0.009978 | 0.830243 | Xp11.23 |

We used the gradient lasso (Least Absolute Shrinkage and Selection Operator) algorithm to

fit a prediction model based on Cox’s proportional hazards model for DFS using the probes with p<0.01.(Sohn I, Kim J, Jung SH, Park C. Gradient lasso for Cox proportional hazards model. Bioinformatics 2009;25:1775-81, Cox D. Regression Models and Life Tables (with Discussion). JR Stat SocSer B 1972;34:187-220).

**Figure S2.** Gradient Lasso algorithm

1. Initialize: $\mathbf{\beta}=0$ and $k=0.$
2. Starting from two consecutive addition steps, proceed deletion and addition steps alternatingly until convergence
3. Addition Step:
4. Let $\nabla C(\mathbf{\beta})$ be the gradient of $C(\mathbf{\beta})$.
5. Compute the gradient $\nabla C(\mathbf{\beta}).$
6. Find the $\hat{j}$ maximizing $\left|\partial C(\beta)/\partial\beta_{j}\right|$for $j(=1,\ldots,m)$and $\hat{r}=-s\times\text{sign}(\partial C(\mathbf{\beta})/\partial\beta_{\hat{j}}$
7. Let $\mathbf{v}$be the p dimensional vector such that its $j-\text{th}$ element $\hat{r}$ and the elements are zeros.
8. Find $\hat{\alpha}=\mathrm{argmin}_{\alpha\in[0, 1]}C((1-\alpha)\mathbf{\beta}+\alpha\mathbf{v}).$
9. Update
10. Deletion Step:
11. Compute $\mathbf{h}_{\sigma}=-\nabla C(\mathbf{\beta}_{\sigma})+\theta_{\sigma}\nabla C(\mathbf{\beta}_{\sigma})^{T}\theta_{\sigma}/\left|\sigma\right|,$

where$\theta_{\sigma}$denotes the sign vector of $\mathbf{\beta}_{\sigma}$and$\left|\sigma\right|$denotes the cardinality of $\sigma.$

1. Let P denote the permutation matrix that collects the non-zero elements of $\mathbf{\beta}$ in the first $\left|\sigma\right|$elementsand$h=\mathbf{P}^{T}\left(\begin{matrix} \mathbf{h}_{\sigma} \\ \mathbf{0} \end{matrix}\right).$
2. Find $\hat{\delta}=\mathrm{argmin}_{\delta\in[0, U]}C(\mathbf{\beta}+\delta\mathbf{h}),$

where$U=\min_{k\in\sigma}\{-\beta_{k}/h_{k}:\beta_{k}h_{k}<0\}.$

1. Update$\mathbf{\beta}=\mathbf{\beta}+\hat{\delta}\mathbf{h}.$


$$k=k+1.$$

3. Return $\mathbf{\beta}$

To evaluate the predictive performance of genes selected by gradient lasso algorithm, we performed leave-one out cross validation (LOOCV) with *de novo* selection of new prognostic genes at each step as suggested by Simon et al (J Natl Cancer Inst, 95:14-18):

Step 1) We partitioned the whole data into a training set of size 431 and a testing set of size 1 by removing the i-*th* sample (i=1,…,432);

Step 2) From the training set, we selected the probes with marginal p-values smaller than 0.01using the univariate Cox regression and standardized each of them to have mean 0 and variance 1. We then applied gradient lasso to the selected probes;

Step 3) For testing set of size 1 of patient *i*, we standardized the expression level of each probe by subtracting the mean and dividing by the standard deviation calculated from the training set. We calculated the predictive score for patient *i* by plugging the standardized expression levels to the prediction model from the training set. The patient *i* was assigned to the high risk group if the score was positive and the low risk otherwise;

Step 4) We repeated steps 2 and 3 for all n=432 cases;

Step 5) We generated Kaplan-Meier curves for the cross validated risk groups (high and low) and calculated the p-value of the log-rank test.

We generated Kaplan Meier plots for DFS based on predicted class assignment of each case during LOOCV procedure (Figure S4).

The above LOOCV shared a large portion of the data set at each discovery group assignment, so that the assignment probabilities are highly correlated through the n=432 leave-one-out procedures. As a result, the log-rank p-value obtained in Step 5 may be biased. For an unbiased comparison between the two groups, we calculated a permutation p-value from 100 permutations of gene expression data as follows:

1. Compute naive p-value (*P*_0_) of the log-rank test from above LOOCV procedure from the original data; (Step 5 above)
2. From the b-th permutation data (b=1,…,B), compute the p-value (*P*_b_) of the log-rank test from Step 5 of the LOOCV procedure;
3. A permutation p-value is calculated by .

The resulting p-value was 0.04 between low and high risk group.

We evaluated the reproducibility of prognostic information by examining number of times each gene was selected by gradient lasso during LOOCV. The 26 probes selected by gradient lasso from all 432 patients are listed in Table S5. Of 26 probes, 16 were selected all 432 times for the model during LOOCV, suggesting that these are robust prognostic genes. Of note is C20orf103 (LAMP5). This gene was the top gene chosen for the final GCPS model during Step 2.

**Table S5.** List of 26 probes included in the prediction model fitted by the whole data set (n=432), their univariate p-values, and the number of times that each probe is included in the prediction models fitted during LOOCV in Step 1.

| PROBE_ID | SYMBOL | P-value | Frequency | Chromosomal Location |
| --- | --- | --- | --- | --- |
| ILMN_2385647 | ALAS1 | 0.005756 | 432 | 3p21.1 |
| ILMN_1713561 | C20orf103 | <0.000001 | 432 | 20p12 |
| ILMN_1787749 | CASP8 | 0.000365 | 432 | 2q33-q34 |
| ILMN_1712088 | CLYBL | 0.000780 | 432 | 13q32 |
| ILMN_1672776 | COL10A1 | 0.000001 | 432 | 6q21-q22 |
| ILMN_1673843 | CST2 | 0.000026 | 432 | 20p11.21 |
| ILMN_1732158 | FMO2 | 0.000001 | 432 | 1q23-q25 |
| ILMN_1811790 | FOXS1 | 0.000000 | 432 | 20q11.21 |
| ILMN_1673548 | HSPC159 | 0.000209 | 432 | 2p14 |
| ILMN_1662824 | MADCAM1 | 0.000956 | 432 | 19p13.3 |
| ILMN_1719543 | MAF | 0.000267 | 432 | 16q22-q23 |
| ILMN_2382679 | REG3A | 0.000232 | 432 | 2p12 |
| ILMN_2071826 | RNF152 | 0.000264 | 432 | 18q21.33 |
| ILMN_1736078 | THBS4 | 0.000001 | 432 | 5q13 |
| ILMN_1757387 | UCHL1 | 0.000201 | 432 | 4p14 |
| ILMN_2093500 | ZBED5 | 0.000081 | 432 | 11p15.3 |
| ILMN_1801205 | GPNMB | 0.000313 | 431 | 7p15 |
| ILMN_1755318 | HIST1H2AJ | 0.000036 | 431 | 6p22-p21.3 |
| ILMN_1729033 | RPL9 | 0.000901 | 431 | 4p13 |
| ILMN_1712506 | DPP6 | 0.007555 | 428 | 7q36.2 |
| ILMN_1769168 | ARL10 | 0.000235 | 421 | 5q35.2 |
| ILMN_1692739 | ISLR2 | 0.001375 | 421 | 15q24.1 |
| ILMN_2316386 | GPBAR1 | 0.000049 | 408 | 2q35 |
| ILMN_1792748 | CPS1 | 0.001224 | 397 | 2q35 |
| ILMN_1665761 | BCL11B | 0.007679 | 378 | 14q32.2 |
| ILMN_1793965 | PCDHGA8 | 0.002196 | 373 | 5q31 |

We next examined the multivariate Cox model in which disease-free survival was evaluated in relation to the classification based on gene signature from LOOCV (low vs high risk), pT stage, number of pathology lymph node metastasis (P NODE) or pathologic stage (AJCC 6th Ed). pT stage was dichotomized into pT1/T2 vs pT3/T4 and P NODE was log-transformed with 0 values replaced by 0.1. Multivariate analysis showed that prognostic gene signature was a significant predictive factor (HR=1.859, 95% CI: 1.367 to 2.530, P = 0.000078) for DFS in gastric cancer patients who received curative resection and adjuvant treatment that was independent of pTstage and P NODE (Table S5). Likewise, prognostic gene signature predicted disease free survival independent from the final pathologic stage with statistical significance (HR=1.773, 95% CI: 1.303 to 2.413, P = 0.000078; Table S6). These data clearly demonstrates that the prognostic gene signature obtained from gene expression analysis of FFPE blocks provides clinically useful prognostic information beyond what is provided by clinical prognostic factors such as TNM staging.

**Table S6**. Multivariate Cox regression analysis results in gene discovery set (n=432)

|  | Hazardratio | 95%CI for hazard ratio | P-value |
| --- | --- | --- | --- |
| Tstage | 2.225 | (1.605,3.085) | 0.000002 |
| Log(PNODE) | 2.129 | (1.612,2.812) | <0.000001 |
| Geneexpression(lowvshigh) | 1.859 | (1.367,2.530) | 0.000078 |
| Multivariate Cox regression analysis-pathologic stage(Pstage) and gene expression based risk groups from leave one out cross validation | | | |
|  | Hazardratio | 95%CIforhazardratio | P-value |
| Pstage | 2.779 | (2.024,3.816) | <0.000001 |
| Geneexpression(lowvshigh) | 1.773 | (1.303,2.413) | 0.000265 |

## 4. Design of focused gene expression assay using nCounter platform

While WG-DASL certainly provided important clues for the potential clinical utility of gene expression based prognostic assay for gastric cancer, especially in stage II patients, WG-DASL assay was far from ideal. In the middle of this study, manufacturer has decided to modify the enzyme used for a key step in the assay and the performance of the assay significantly degenerated since. Eventually WG-DASL assay was discontinued. From the design stage, our intention was to use more robust gene expression analysis platform for clinical assay development using candidate prognostic genes discovered from WG-DASL assay. One potential platform was QRT-PCR assay, but it was too expensive and labor intensive to screen more than several hundred genes using QRT-PCR platform.

In order to develop a robust clinical grade assay that can be performed using degraded RNA from FFPET as starting materials, we decided to use nCounter assay platform. nCounter assay allowed interrogation of up to 800 genes in a single tube reaction using degrade RNA extracted from old archived FFPET samples.

One of the barriers to reduce the discovered prognostic genes into clinical practice is the need for self-normalization within each case, because patients do not come all at once to allow cohort based normalization. For clinical tests such as nCounter assay or QRT-PCR with limited number of probes, one cannot measure all human genes to be able to perform quantile normalization, which is usually used for normalization of microarray gene expression data. It is also know that absolute QRT-PCR signals are much lower when old paraffin blocks are used as starting materials compared using newer materials as illustrated by Cronin et al (AJPathol 2004;164:35-42). One way to overcome this problem is to normalize the data using internal reference genes as shown by Cronin et al. However for each tumor types, internal reference genes need to be carefully selected. Using WG-DASL data we were able to select a set of 50 genes that has least variability among different cases and has no prognostic value. We tested the correlation between quantile normalized and self-normalized data in our WG-DASL dataset. Hazard rates based on these two normalization methods are plotted in Figure S5. The data clearly shows that there is a good correlation between the two methods. Of these 50 genes, 2 genes did not show acceptable analytical performance in nCounter assay, and therefore 48 genes were used to normalize nCounter assay data (Table S7).

We designed anCounter probes set based on candidate prognostic genes from WG-DASL data and screened 96 cases to examine concordance of expression levels between DASL and nCounter probes. 82 DASL originated nCounter probes that showed good correlation with DASL data were combined with other genes of interest (cancer genes, kinase genes, GPCR genes) to design the second set of nCounter probes to be used for the validation study.

**Table S7. List of reference genes for nCounter assay**

| *Name* | *Accession* |
| --- | --- |
| ABHD2 | NM_007011.6 |
| AFF4 | NM_014423.3 |
| ANKRD36B | NM_025190.3 |
| ARMC1 | NM_018120.3 |
| ASPH | NM_032466.3 |
| C11orf24 | NM_022338.3 |
| C13orf31 | NM_001128303.1 |
| C1RL | NM_016546.1 |
| CHPT1 | NM_020244.2 |
| CLIC2 | NM_001289.4 |
| COPS8 | NM_006710.4 |
| CRY2 | NM_001127457.1 |
| ERGIC1 | NM_020462.1 |
| ERMP1 | NM_024896.2 |
| FOXO4 | NM_005938.2 |
| GIMAP8 | NM_175571.2 |
| GLRX | NM_002064.2 |
| HSFX1 | NM_016153.2 |
| ILF3 | NM_001137673.1 |
| ING4 | NM_001127582.1 |
| KIAA0182 | NM_014615.2 |
| LMAN2 | NM_006816.2 |
| MADD | NM_130476.2 |
| MAPKAPK2 | NM_004759.3 |
| MID1 | NM_033291.1 |
| MTR | NM_000254.2 |
| MUS81 | NM_025128.4 |
| NAT14 | NM_020378.2 |
| NBPF15 | NM_001170755.1 |
| PHF20L1 | NM_024878.1 |
| PPIA | NM_021130.2 |
| PRKAA1 | NM_006251.5 |
| PTK7 | NM_152881.2 |
| PTN | NM_002825.5 |
| RAB24 | NM_001031677.2 |
| RAP2C | NM_021183.3 |
| RCSD1 | NM_052862.3 |
| SFRS12 | NM_001077199.1 |
| SFRS17A | NR_027383.1 |
| SUMO1 | NM_003352.4 |
| SYNM | NM_015286.4 |
| TADA3 | NM_006354.2 |
| TBC1D9B | NM_198868.2 |
| TERF2IP | NM_018975.2 |
| TMBIM1 | NM_022152.4 |
| USF2 | NM_003367.2 |
| ZNF146 | NM_007145.2 |
| ZNF33A | NM_006954.1 |

**Figure S3.** Correlation between hazard ratios of prognostic genes based on quantile normalization and self-normalization using WG-DASL assay

## 5. nCounter assay and quality control

Using NanoStringnCounter™ technologies (Geiss GK, Bumgarner RE, Birditt B, et al. Direct multiplexed measurement of gene expression with color-coded probe pairs. Nat Biotechnol 2008;26:317-25.), we designed and synthesized probe sets that hybridized specifically to each mRNA target. This code set contained a biotinylated capture probe and a reporter probe attached to a color-barcode tag, according to the nCounter™ code-set design. We hybridized 200ng of RNA for 18hrs at 65°C with the code set and loaded into the nCounterPrepstation followed by quantify target mRNA in each sample using the nCounter Digital Analyzer. We excluded the outlier samples based on following quality control criteria: a normalization factor based on the sum of the positive control counts larger than 3 fold change and the number of probes detected based on the maximum of negative controls smaller than 360. The count of the probes were transformed by logarithm with base 2 and normalized using the 48 internal control genes (Table S6).

## 6. Selection of cut-off for Gastric Cancer Prognostic Score (GCPS)

**Figure S4.** DFS of discovery set according to quartile values of GCPS. Red=1^st^ quartile (5-year DFS = 0.87), Black=2^nd^ quartile (5-year DFS = 0.86), Green=3^rd^ quartile (5-year DFS = 0.72), Blue=4^th^ quartile (5-year DFS = 0.51).


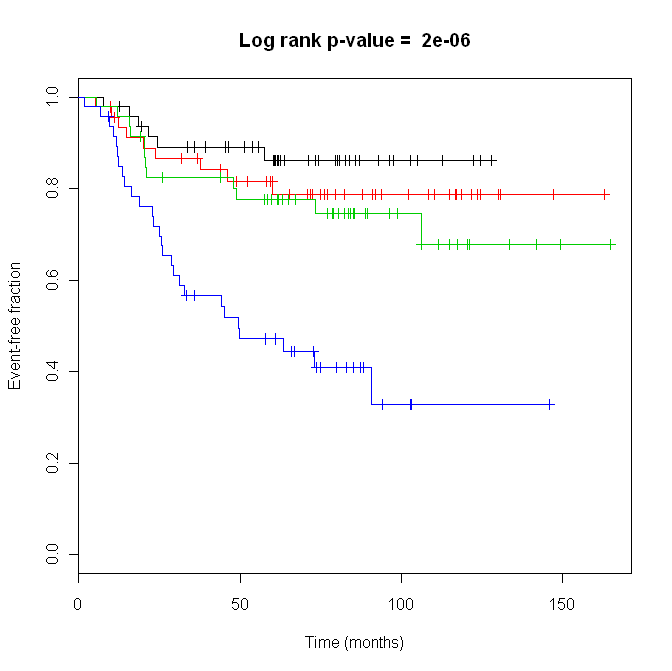


Because of this non-linearity, we performed cut-off analysis by calculating p-values for each possible cut-offs for GCPS. The result is shown in Figure S6. This plot shows that optimal cut off is around top 15-25 percentile. We chose to use 25% as a cut-off for validation study. This corresponds to GCPS of 0.2205.

**
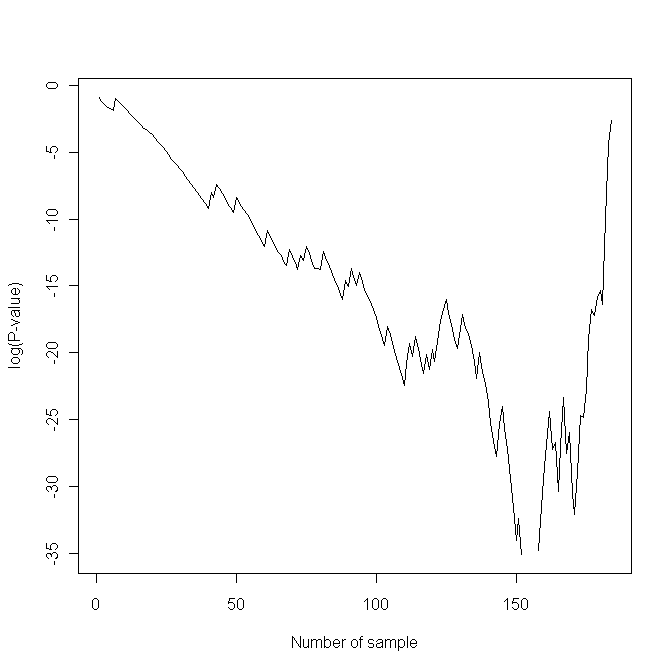
Figure S5*.*** Cut-point analysis for GCPS.

**Figure S6*.*** KM plot of DFS of discovery stage II cohort based on optimized GCPS-g1 cut-off (0.2205)


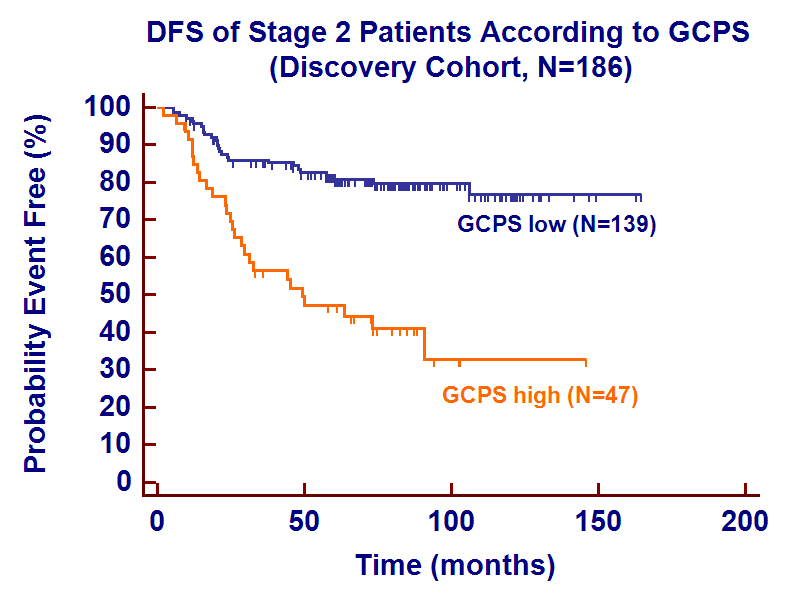


## 7. Distribution of GCPS between discovery set and validation set.

One of the parameters we can use to test the analytical performance of GCPS is to check the distribution of GCPS values between discovery and validation set. Figure S7 shows that GCPS distribution is nearly identical between the two cohorts. While the pre-defined cut-off for defining high-risk (0.2205) classified 25% of discovery set into high-risk, 22.7% were classified as high-risk in the validation set.

**
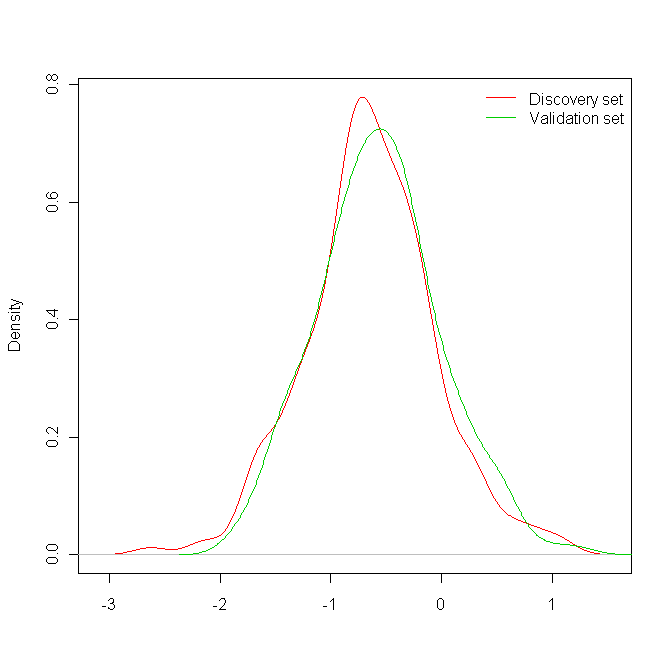
Figure S7.** Distribution of GCPS within the discovery and validation set.

**Figure S8.** GCPS: intestinal vs diffuse type


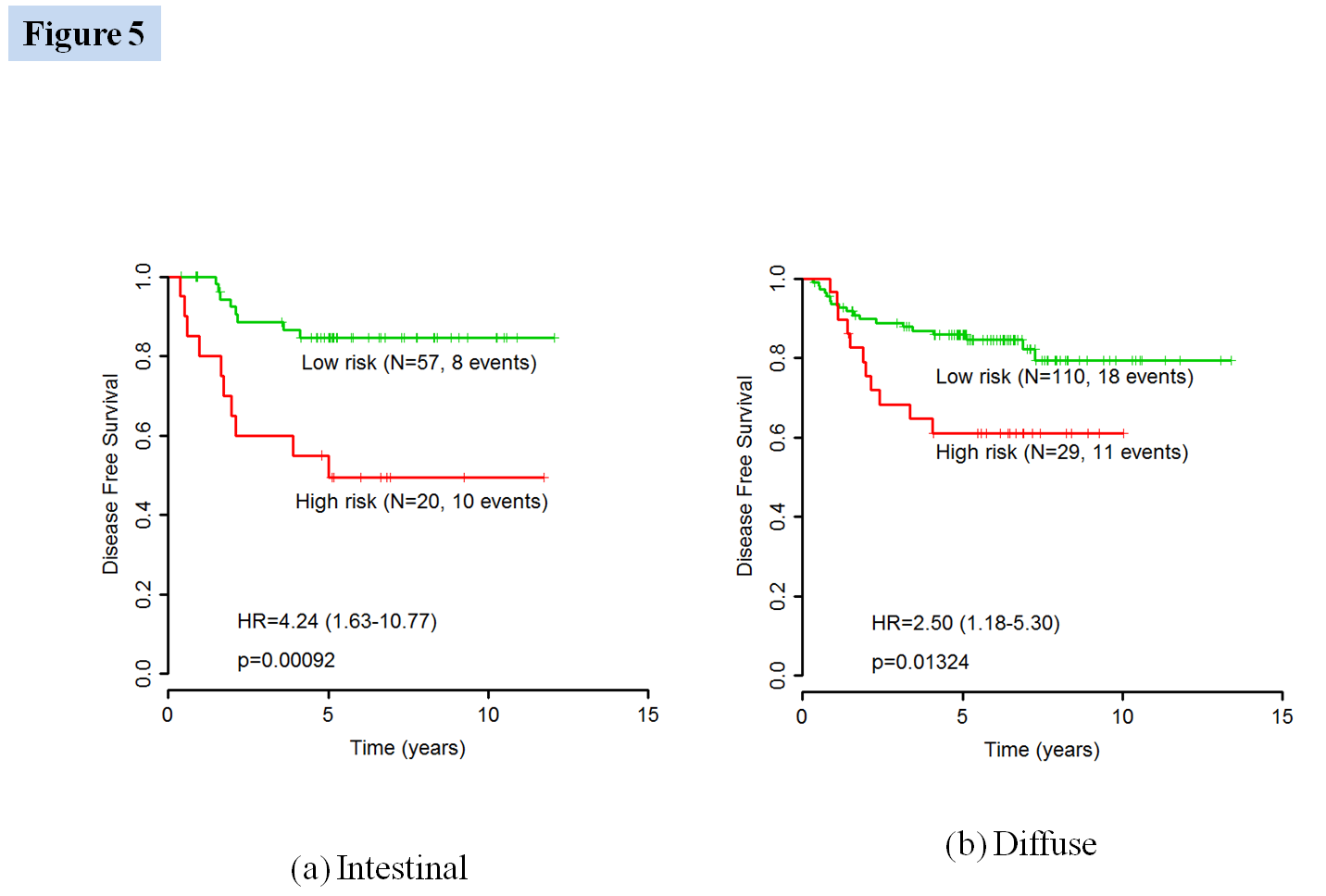


## Testing of clinical utility of GCPS-g2 in patients treated with surgery only

Having validated the performance of gene expression based prognostic indexes for prognostication of stage II gastric cancer patients, we then combined data from all stage 2 patients (N=402) for second generation GCPS (GCPS-g2) building and cut-off determination that can be applied to the phase 4 clinical utility study. Our intent was to utilize as much data as possible to build a stable algorithm before final validation step.

Figure S11 shows the results of LOOCV within the entire phase 2 study cohort of 402 patients. Again, the developed gradient lasso algorithm was robust in identifying high-risk patients with 5 year DFS of 51% compared to 87% of the lowest risk group.

**Figure S9*.*** *DFS of stage II patients (n=402) based on quartiles of risk scores from LOOCV of gradient lasso prognostic algorithm.*


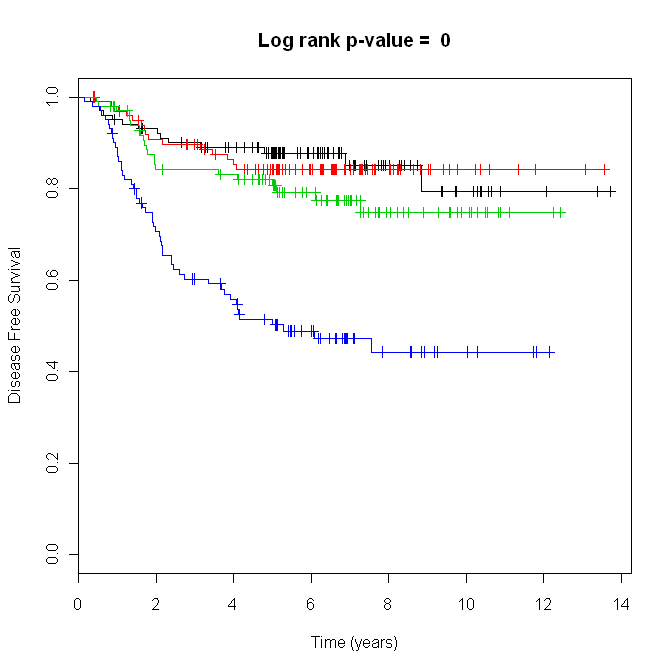


We used the gradient lasso algorithm to develop a GCPS-g2 based on the Cox’s proportional hazards model for DFS.

Table S8 lists the 13 probes that are included in the prediction model fitted using the phase 2,3 data set (n=402). The risk score of a patient with expression data for the 13 probes is calculated as where denotes the regression coefficient of probe j in the fitted prediction model.

We estimated the cut off for the first quartile (Q1=-0.9843) and the cut off for the third quartile (Q3=-0.4478) of the distribution of risk scores from the phase 2 data set of 402 stage 2 patients. Applying them to our final validation set of 306 patients, we assigned the patients with risk scores smaller than Q1 to the low risk group and those with risk scores larger than Q3 to the high risk group.

**Table S8*.*** *List of nCounter probes and their regression estimates from the phase 2 data set (n=402) to calculate second generation Gastric Cancer Prognostic Score (GCPS-g2) . * denotes genes also included in GCPS-1g*

| Gene Symbol | Regression estimate |
| --- | --- |
| ADRA2C | -0.0156 |
| C20orf103* | 0.1082 |
| CLIP4* | 0.3891 |
| CSK | -0.6654 |
| FZD9 | -0.0829 |
| GALR1 | -0.0509 |
| GRM6 | -0.0244 |
| INSR | 0.0251 |
| LPHN1 | -0.0126 |
| LYN | -0.0012 |
| MATN3* | 0.2134 |
| MRGPRX3 | -0.0009 |
| NOX4* | 0.0951 |

We applied predefined cut-point for GCPS-g2 to classify surgery only patients cohort into low- or high-risk and generated KM plots shown in Figure S11. The classification based on GCPS-g2 resulted in low and high risk groups with statistically significant difference in DFS (HR= 2.131, 95% CI:1.428-3.180, p=0.00021, N=300).

**Figure S10.**

**
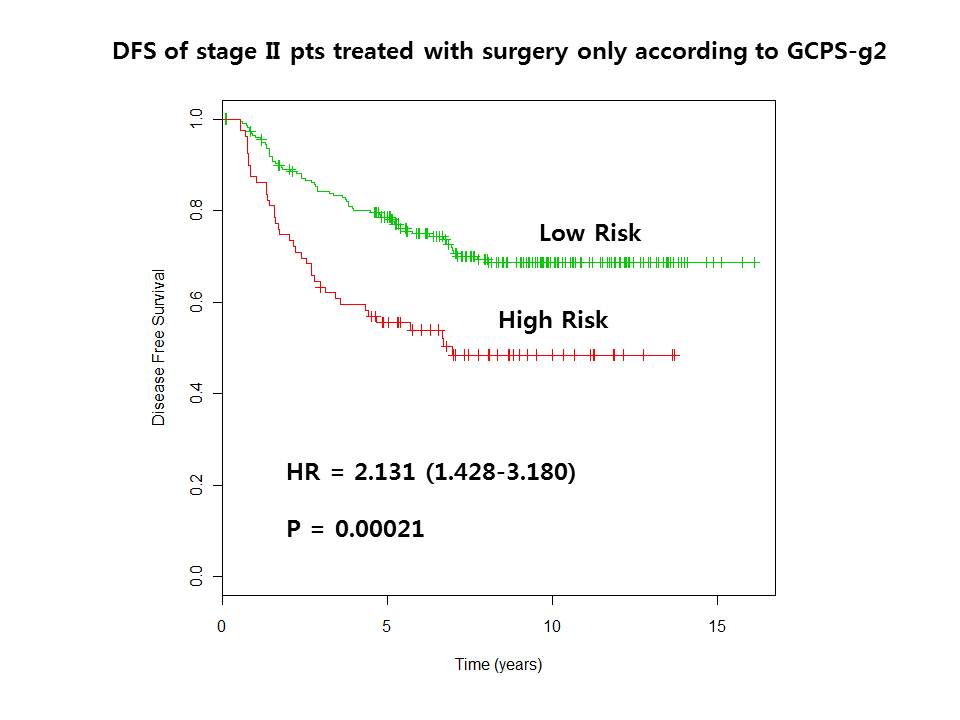
**

**Figure S11.**

## Gastric cancer validation study protocol signed

**Figure S12. Gastric cancer validation study protocol signed**


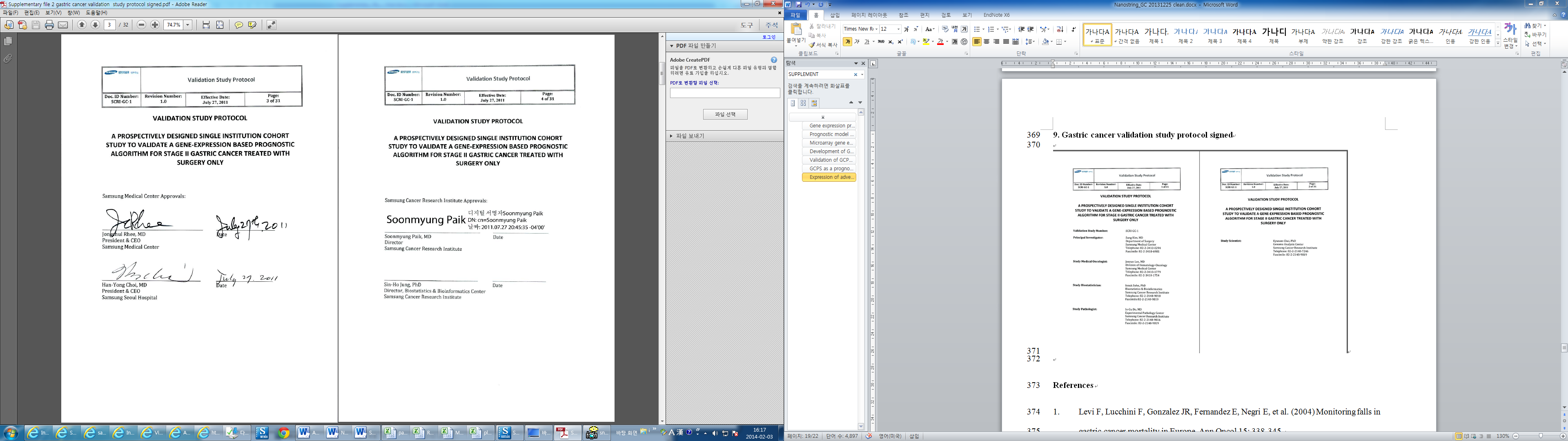

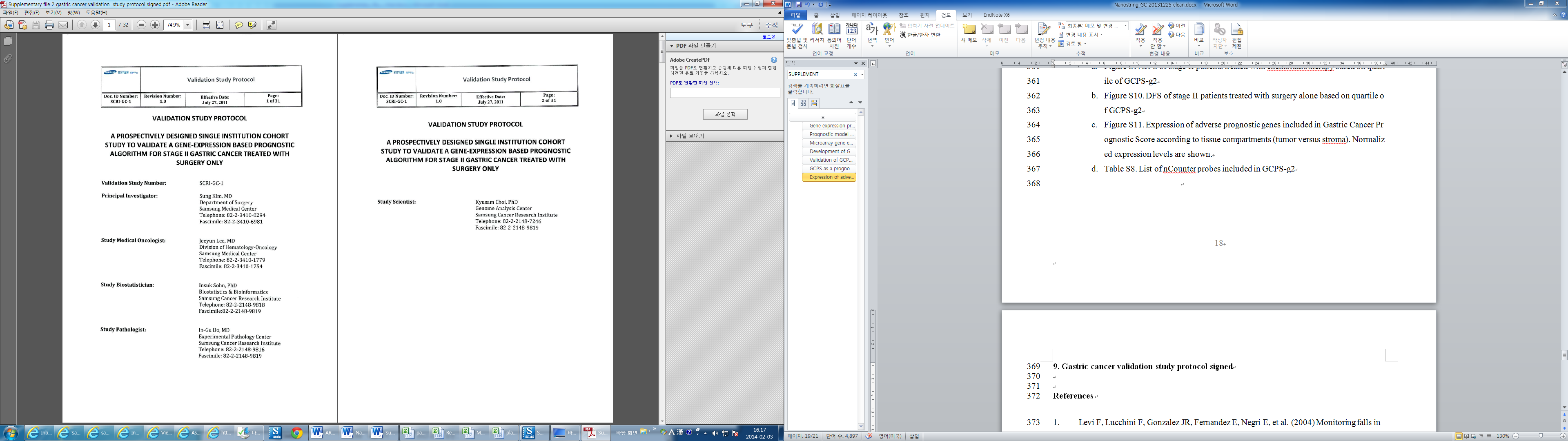


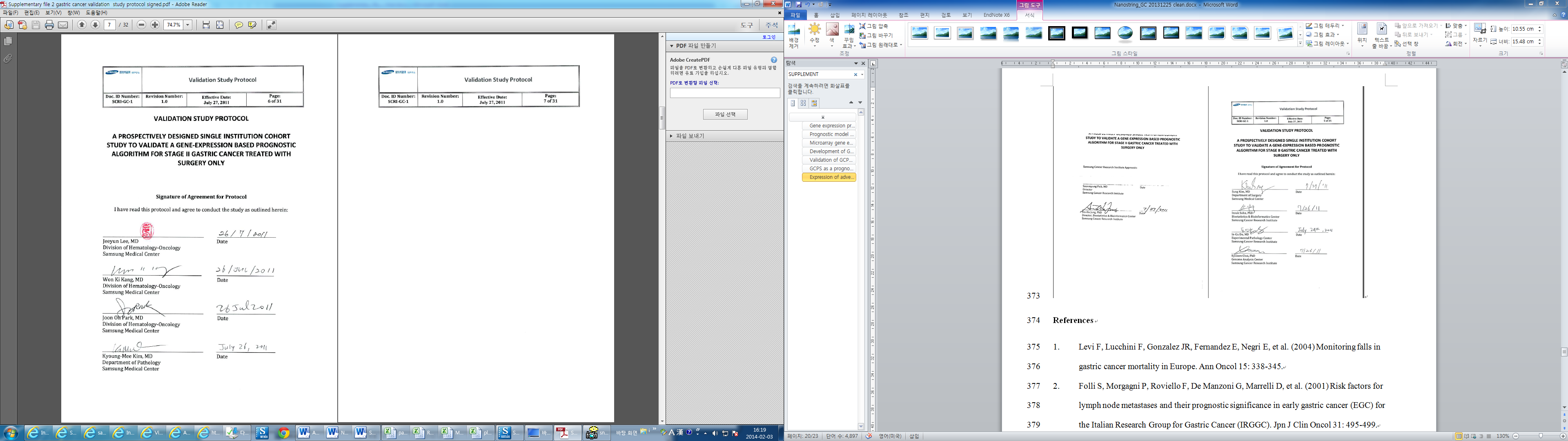

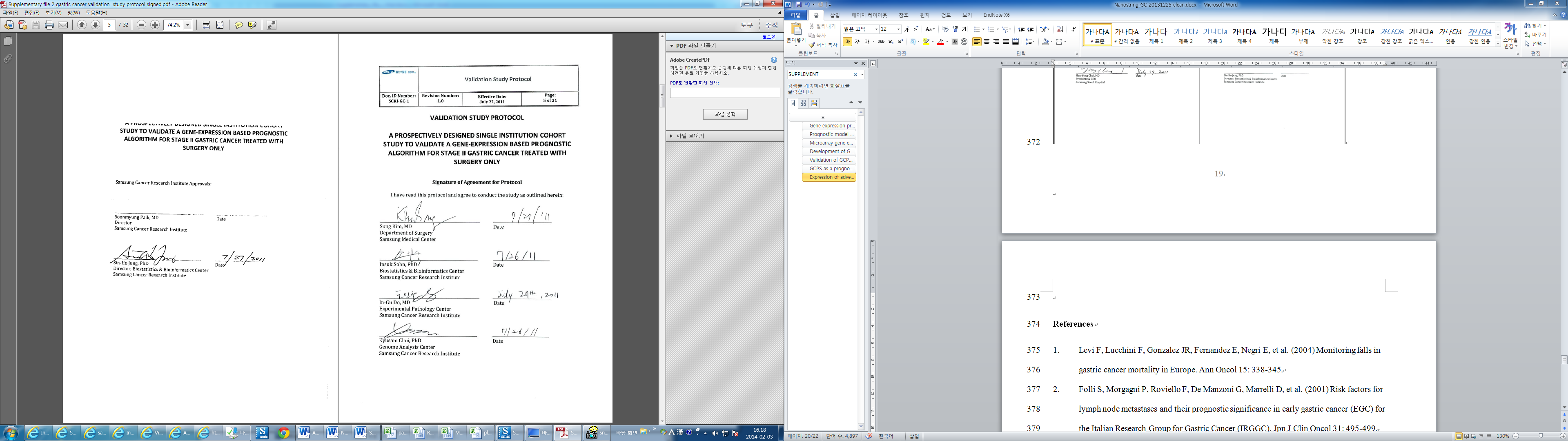

Supplement: File S1 — 1. Patient characteristics of study cohorts at each step (Table S1). a. Table S1. Patients characteristics. 2. Clinical and pathological characteristics of cases examined at each phase. 3. Detailed description of the discovery step using WG-DASL assay (step 1). a. Figure S1. QA of WG-DASL data. b. Table S2. Comparison of FISH and IHC results for HER2 status in gastric cancer in Step 1. c. Table S3. List of probes that are differentially expressed between HER2-positive and HER2- negative patient groups in Step 1. d. Table S4. List of all probes with univariate p-values<0.01 in Step 1. e. Figure S2. Gradient Lasso algorithm. f. Table S5. List of 26 probes included in the prediction model fitted by the whole data set (n = 432). g. Table S6. Multivariate Cox regression analysis results in gene discovery set (n = 432). 4. Design of focused gene expression assay using nCounter platform. a. Table S7. List of reference genes for nCounter assay. b. Figure S3. Correlation between hazard ratios of prognostic genes based on quantile normalization and self-normalization using WG-DASL assay. 5. nCounter assay and quality control. 6. Selection of cut-off for Gastric Cancer Prognostic Score (GCPS(Figure S4, S5, & S6). a. Figure S4. DFS according to each quartiles of GCPS-g1. b. Figure S5. Cut-point analysis for GCPS-g1. c. Figure S6. DFS according to optimized cut-point of GCPS-g1. 6. Distribution of GCPS between discovery set and validation set. a. Figure S7. Distribution of GCPS-g1 within the discovery and validation set. b. Figure S8. GCPS: intestinal vs diffuse type. 7. Testing of clinical utility of GCPS-g2 in patients treated with surgery only. a. Figure S9. DFS of stage II patients treated with chemoradiotherapy based on quartile of GCPS-g2. b. Figure S10. DFS of stage II patients treated with surgery alone based on quartile of GCPS-g2. c. Figure S11. Expression of adverse prognostic genes included in Gastric Cancer Prognostic Score according to tissue compartments (tumor ve [file pone.0090133.s001.docx]
